# Design and Characterization of Gold Nanorod Hyaluronic Acid Hydrogel Nanocomposites for NIR Photothermally Assisted Drug Delivery

**DOI:** 10.3390/gels12010088

**Published:** 2026-01-19

**Authors:** Alessandro Molinelli, Leonardo Bianchi, Elisa Lacroce, Zoe Giorgi, Laura Polito, Ada De Luigi, Francesca Lopriore, Francesco Briatico Vangosa, Paolo Bigini, Paola Saccomandi, Filippo Rossi

**Affiliations:** 1 Department of Chemistry, Materials and Chemical Engineering “Giulio Natta”, Politecnico di Milano, Via Luigi Mancinelli, 7, 20131 Milan, Italy; alessandro.molinelli@polimi.it (A.M.); elisa.lacroce@polimi.it (E.L.); zoe.giorgi@polimi.it (Z.G.); francesca.lopriore@polimi.it (F.L.); francesco.briatico@polimi.it (F.B.V.); 2 Department of Mechanical Engineering, Politecnico di Milano, Via Giuseppe La Masa, 1, 20156 Milano, Italy; leonardo.bianchi@polimi.it (L.B.); paola.saccomandi@polimi.it (P.S.); 3 Consiglio Nazionale Delle Ricerche, CNR-SCITEC, Via Gaudenzio Fantoli, 16/15, 20138 Milan, Italy; laura.polito@scitec.cnr.it; 4 Department of Biochemistry and Molecular Pharmacology, Istituto di Ricerche Farmacologiche Mario Negri IRCCS, Via Mario Negri, 2, 20156 Milan, Italy; ada.deluigi@marionegri.it (A.D.L.); paolo.bigini@marionegri.it (P.B.)

**Keywords:** hydrogel, hyaluronic acid, gold nanorods, stimuli-responsiveness

## Abstract

The combination of gold nanoparticles (AuNPs) with hydrogels has drawn significant interest in the design of smart materials as advanced platforms for biomedical applications. These systems endow light-responsiveness enabled by the AuNPs localized surface plasmon resonance (LSPR) phenomenon. In this study, we propose a nanocomposite hydrogel in which gold nanorods (AuNRs) are included in an agarose–carbomer–hyaluronic acid (AC-HA)-based hydrogel matrix to study the correlation between light irradiation, local temperature increase, and drug release for potential light-assisted drug delivery applications. The gel is obtained through a facile microwave-assisted polycondensation reaction, and its properties are investigated as a function of both the hyaluronic acid molecular weight and ratio. Afterwards, AuNRs are incorporated in the AC-HA formulation, before the sol–gel transition, to impart light-responsiveness and optical properties to the otherwise inert polymeric matrix. Particular attention is given to the evaluation of AuNRs/AC-HA light-induced heat generation and drug delivery performances under near-infrared (NIR) laser irradiation in vitro. Spatiotemporal thermal profiles and high-resolution thermal maps are registered using fiber Bragg grating (FBG) sensor arrays, enabling accurate probing of maximum internal temperature variations within the composite matrix. Lastly, using a high-steric-hindrance protein (BSA) as a drug mimetic, we demonstrate that moderate localized heating under short-time repeated NIR exposure enhances the release from the nanocomposite hydrogel.

## 1. Introduction

Over recent decades, controlled drug delivery systems (DDSs) have attracted growing interest due to their significant advantages over conventional dosage forms [1]. Stimuli-responsive moieties have been employed to design smart DDSs, offering precise spatiotemporal control over drug release in response to exogenous or endogenous triggers [2,3]. The employed materials include inorganic and organic nanoparticles (NPs) [4,5], natural and synthetic polymers, whose responsiveness arises from their architecture, composition, and functional groups [6,7], which can be tailored at a molecular level to suit specific applications [8]. While the fundamental principles governing stimuli-responsive drug release are well established, limited research has focused on the development of well-defined model platforms that allow systematic investigation of structure–property–function relationships, rather than on the discovery of new release mechanisms per se. A more recent approach for smart DDS design involves the combination of hydrogel with responsive NPs. Hydrogels are characterized by unique properties such as water retention, biocompatibility, tunable degradability, and porosity [9]. Their three-dimensional crosslinked network, obtained through either chemical or physical interactions, acts as a drug depot and enables drug release through different mechanisms (such as diffusion, swelling, and either surface or bulk erosion). However, they often lack mechanical strength, and possess limited intrinsic stimuli-responsiveness, if not properly formulated using engineered stimuli-responsive constituents [10]. NPs’ inclusion within the hydrogel matrix represents a compelling strategy to address these shortcomings. In this way, the final systems are provided with improved mechanical strength, and responsiveness to a variety of stimuli, according to the specific NPs employed [11,12,13]. Meanwhile, the hybrid platform acts as NPs support, confining their properties and limiting their release, clearance, or bioaccumulation [14,15]. Between the various stimuli, light has emerged due to its minimal invasiveness, short treatment duration, and rapid patient recovery [16] and has been explored in laser ablation of tumors, drug delivery, and imaging [17,18]. A near-infrared (NIR) wavelength (ranging from 700 to 1100 nm) provides several advantages over UV or visible ones, thanks to deeper tissue penetration, lower phototoxicity, and reduced absorption by biological fluids and tissue chromophores (such as melanin and hemoglobin), resulting in a safe tool for biomedical applications [19,20,21].

Gold nanoparticles (AuNPs) can be used as moieties for designing light-responsive DDSs, thanks to their optical properties originated by the localized surface plasmon resonance (LSPR). This phenomenon is given by the collective oscillation of AuNPs surface electrons (plasmons) upon interaction with light, with wavelengths higher than AuNPs’ dimensions [22,23]. As a consequence, light irradiation in resonance with AuNPs’ plasmonic peak leads to efficient light-to-heat conversion in AuNPs’ vicinity [24]. AuNPs’ properties have been exploited for drug delivery, tumor ablation, biosensing, and targeted therapies [25,26]. Thanks to their geometry, gold nanorods (AuNRs) are characterized by two different plasmonic modes, respectively associated with the longitudinal and transversal dimensions. The longitudinal plasmonic peak can be adjusted within the NIR region by slight modification of their aspect ratio (length vs. width) through chemical synthesis [27]. Despite these properties, AuNPs’ limitations, such as their non-biodegradability, bioaccumulation, cytotoxicity, low stealth properties, and poor colloidal stability (if not properly functionalized), still hinder their medical applications [14,28]. Additionally, drug loading is constrained to direct surface interaction or through chemical linkers, as the metallic gold core does not support internal encapsulation [29]. By embedding AuNPs within hydrogels, light-responsive composite materials can be obtained, while mitigating the single-system drawbacks. Composite systems can improve AuNPs’ dispersion stability and can entrap AuNPs within a specific space [30]. In parallel, they can act as depots for encapsulated bioactive molecules, which can be released with beneficial effects compared to classical administration methods. Additionally, the hydrogel matrix would inhibit the direct interaction of the AuNPs with the external biological tissues, providing protection from the external environment [12]. Thanks to these features, AuNPs’ optical properties can be restricted within the hydrogel volume, with superior advantages for applications such as hyperthermia and light-mediated drug delivery. However, limited studies have been presented on the correlation between light-induced heating and drug release as well as precise thermal mapping within the nanocomposites [15]. Although photothermally assisted release from AuNPs-based systems has been previously reported, most studies focus on demonstrating release enhancement, while less attention has been devoted to systematically correlating local temperature evolution, spatial heat distribution, and release kinetics within confined hydrogel networks. In hydrogel design strong attention should be given not only to the components, that should be biodegradable and biocompatible, but also the manufacturing procedures that should involve facile and reproducible steps in order to guarantee reliable production and avoid problems in the scale up. Moreover, sterilization should also be taken into account, being very challenging in hydrogel systems due to their high content of water and sensitivity to heat.

Based on these observations, the study aims to propose a proof-of-concept platform demonstrating how AuNR-integrated hydrogel can be engineered to achieve light-assisted drug release. Importantly, this work does not aim to demonstrate therapeutic efficacy or disease-specific applicability, but rather to establish a reproducible proof-of-concept material platform for mechanistic investigation of light-induced thermal effects on molecular release under NIR light irradiation. Accordingly, ternary agarose–carbomer–hyaluronic acid (AC-HA) hydrogel formulations have been investigated as a function of variation of hyaluronic acid molecular weights and ratios. Then AuNRs were encapsulated into the matrix to evaluate the nanocomposite hydrogel properties as a function of AuNR concentration. Successively, NIR laser irradiation was employed to study the system photothermal performances and to evaluate the maximum temperature increase attained at a fixed AuNR concentration. Bovine serum albumin (BSA) was used as protein drug mimetics for light-assisted release tests to correlate the generated heat with the drug delivery enhancement of the system, while monitoring the AuNRs/AC-HA temperature evolution in real time using a fiber Bragg grating (FBG) sensor array.

The FBG allowed precise and low-invasive spatiotemporal thermal profiling during the irradiation time, with the advantage of precisely evaluating the temperature inside the AuNR/AC-HA hydrogel composite, without damaging the structure. FBG sensors were adopted for the specific application, due to miniaturized dimension, immunity from electromagnetic interferences, and the possibility of performing quasi-distributed temperature measurements, thanks to multiple sensing points within a single fiber and multiplexing capability [16,31]. Although they have advantages over commonly used techniques for temperature monitoring, e.g., thermocouple and thermal imaging, limited applications have been developed using FBG sensors in the field of light-enhanced drug delivery in hydrogel containing Au-based NPs. This approach enables a detailed level of spatially resolved in situ thermal characterization that is rarely achieved in hydrogel-based photothermal systems, providing critical insight into the relationship between localized heat generation by AuNRs and release behavior, compared to the previous methods.

## 2. Results and Discussion

### 2.1. Gold Nanorod Synthesis and Characterization

**Figure 1:**
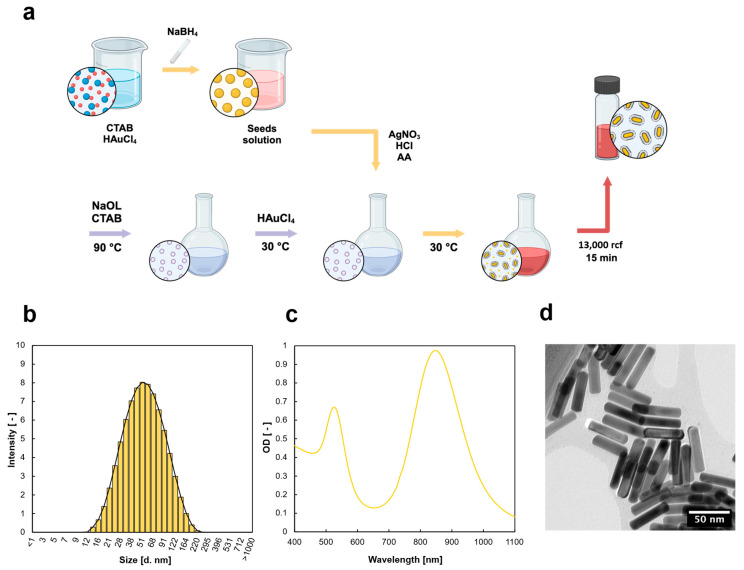
AuNRs synthesis and characterization. (**a**) Schematic illustration of the synthetic steps employed to obtain purified AuNRs. (**b**) DLS measurements of AuNRs with histogram distribution after purification. (**c**) UV-Vis absorbance spectrum of the AuNR solution. (**d**) Transmission electron microscopy (TEM) acquisition of the synthesized AuNRs sample.

AuNRs were synthesized with an optimized procedure (see Section 2.1) to obtain a longitudinal UV-Vis plasmonic peak in the NIR window. The synthetic procedure followed a two-step method in which gold nanocrystals are prepared using a strong reductant (NaBH_4_) and used as starting nuclei for AuNRs formation (Figure 1a). It has been previously found that the presence of Ag^+^ ions and a mild reducing agent, such as ascorbic acid, are crucial for high-yield AuNRs synthesis, together with cetyltrimethylammonium bromide (CTAB) [32]. CTAB is known to preferentially bind to the longitudinal side of the particle, determining the AuNRs’ growth along the longitudinal axis. In addition to the synthetic purpose, it also acted as a stabilizer for the colloidal system, preventing AuNRs aggregation. Due to the presence of high CTAB concentrations, an additional purification step was needed in our procedure to remove the excess surfactant, which influenced the detected AuNRs’ size in dynamic light scattering analysis, due to CTAB’s micellization ability. The synthesized AuNRs were characterized by a hydrodynamic diameter (d_h_) of 58 nm and a positive surface charge of around +33 mV, both measured through dynamic light scattering (DLS) as reported in Figure 1b. The absorbance spectrum (Figure 1c) outlined the presence of two different peaks, one associated with the longitudinal plasmon oscillation (at λ = 527 nm), and the second one in the NIR region (at λ = 847 nm), corresponding to the longitudinal oscillation of the electrons. The correct and efficient synthesis of the desired NPs shape was assessed through transmission electron microscopy (TEM) acquisition (Figure 1d), from which it was possible to appreciate that the synthesized AuNRs have dimensions of approximately 50 × 12 nm.

### 2.2. AC-HA Hydrogel Formulation and Characterization

**Figure 2:**
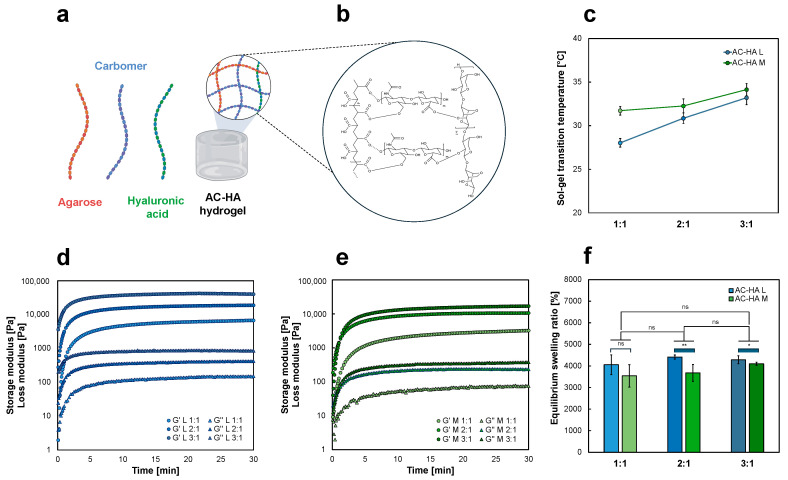
AC-HA hydrogel gelification and characterization. (**a**) Schematic representation of the hydrogels with the three components in the formulation, agarose, carbomer, and hyaluronic acid, respectively, and (**b**) magnification of a possible chemically crosslinked AC-HA network. (**c**) Sol–gel transition temperature (crossover point between G′ and G″ as a function of temperature) obtained through temperature sweep test. (**d**) Gelification kinetics analyzed with time sweep tests representing the evolution of G′ and G″ plateaus over time of all the dilutions tested (1:1, 2:1, and 3:1 respectively shown with increasing intensity of color) for AC-HA-L and (**e**) AC-HA-M formulations. (**f**) Equilibrium swelling behaviors of the AC-HA-L (blue) and AC-HA-M (green) at three different dilution ratios for the formulations. Results presented as mean ± SD, * *p* < 0.05 and ** *p* < 0.01. The values reported were evaluated with measurements run at least in triplicates.

The hydrogel was based on a formulation selected from previous works, composed of FDA-approved materials (agarose–carbomer, AC) and enriched with HA, which was the most abundant polymer in the employed formulation [33]. HA was not only selected for its biocompatibility but also for its polyanionic nature in physiological conditions [34], which would promote electrostatic interaction with CTAB-stabilized AuNRs and macromolecular payloads [35]. This polymer additionally offers the possibility of tuning hydrogel mesh and degradation with modification of its molecular weight [33]. HA is advantageous compared to alternative polymers such as alginate or gelatin, as the latter would require ionic crosslinking. Moreover, these polymers generally exhibit limited NPs retention, and they offer poor mechanical properties and augmented physiological degradation [36]. Additionally, HA presents free carboxyl groups, which are essential for the selected gelification mechanism. Indeed, matrix crosslinking has been obtained through a polycondensation reaction between carboxylic groups (-COOH) and hydroxyl groups (-OH) respectively present in the carbomer, HA, and agarose repeating units (sketch in Figure 2a,b).

The reaction was driven by microwave irradiation, which provided efficient and uniform heating to the reactive mixture for the ester bond formation between the hydrogel constituents at 80 °C. The advantage of this reaction consisted of the absence of any organic solvents, any crosslinkers, or catalysts. No polymer modifications were needed to form the hydrogel matrix, and no washing steps were employed to remove toxic residues. In this way, HA has been crosslinked, without using HA-modified gelification methods, e.g., photo-crosslinking or external crosslinkers. Although chemical modifications may introduce additional properties to the polymers, the process would require purification and post-purification analysis, increase the complexity of the hydrogel formulation strategy, and potentially introduce toxicity sources to the otherwise safe biopolymers. An additional advantage is represented by the formation of chemically crosslinked hydrogels within a 1 min reaction and around 5 min of cooling. This is faster than other methods recently published, which require longer times and several polymer chemical modifications [37,38,39]. By employing this method, molecules, such as drugs, or colloidal systems, e.g., NPs dispersions, could be easily encapsulated inside the three-dimensional network before the complete gelation takes place.

The hydrogels were obtained after sol–gel transition by cooling down, and the gelification was assessed in terms of IR spectroscopy, demonstrating the formation of the ester bonds (as reported in Figure S1, Supporting Information). The hydrogels were formulated using different HA molecular weights (specifically 10 kDa, HA-L, and 40 kDa, HA-M) at different polymer concentrations to investigate their impact on the system’s macroscopical properties. In this regard, three different *v*/*v* dilution ratios—namely 1:1, 2:1, and 3:1—were used, where the first number represents the pre-gel solution and the second refers to the diluent (Table 1).

#### 2.2.1. Gelation Kinetics Characterization

The gelification time was analyzed through inverted tube tests, showing that the gel rapidly formed at room temperature a few minutes after the microwave reaction (as reported in Table S1, Supporting Information). Temperature sweep tests reported in Figure 2c showed the crosslinking formation at the point of crossover between the storage modulus (G′) and the loss modulus (G″). The system behaved as a liquid (G″ > G′) at temperatures higher than the crossover point, whilst having a solid-like behavior (G′ > G″) by cooling down. The sol–gel transition temperatures values recorded were in the range 28–34 °C, as shown in Figure 2c (additional data reported in Figure S2, Supporting Information). Real-time gelification kinetics have been further studied using a time sweep test, demonstrating G′ > G″ throughout the observed time window.

The curves reached a plateau after around 5–8 min, regardless of the formulation, as reported in Figure 2d,e. Thus, neither dilution ratio nor HA molecular weight had a significant influence on the gelation time, gelation kinetics, and gelation temperature.

#### 2.2.2. Swelling Behavior Characterization

Swelling tests performed at room temperature revealed that the different formulation ratios did not significantly change the dried hydrogels to reabsorb water, both in terms of kinetics (Figure S3, Supporting Information) and equilibrium swelling ratio (*p* > 0.05). Indeed, the gels reached an equilibrium swelling ratio in a range of 3500–4500% after around 5 h, regardless the formulation, as shown in Figure 2f. A slightly higher equilibrium swelling ratio (4000–4400%) was observed for HA-L compared to HA-M (3800–4300%). The data were in line with previously reported results in the literature [33], although the reported differences are not strongly significant (1:1, *p* > 0.05; 2:1, *p* < 0.01; 3:1, *p* < 0.05). Although uncrosslinked HA typically showed higher swelling ratios with increasing molecular weight in past studies [40], the observed behavior could be attributed to two possible factors: (1) HA in our study was crosslinked; and (2) the higher amount of -OH and -COOH reactive groups in the HA-M repeating units may have contributed to additional crosslinking. As a result, a slightly lower equilibrium swelling ratio was observed, due to the possibly denser hydrogel matrix. Moreover, longer HA chains may enhance the physical entanglement between the polymeric networks, contributing to reducing the equilibrium swelling ratios [41].

#### 2.2.3. Rheological Characterization

**Figure 3:**
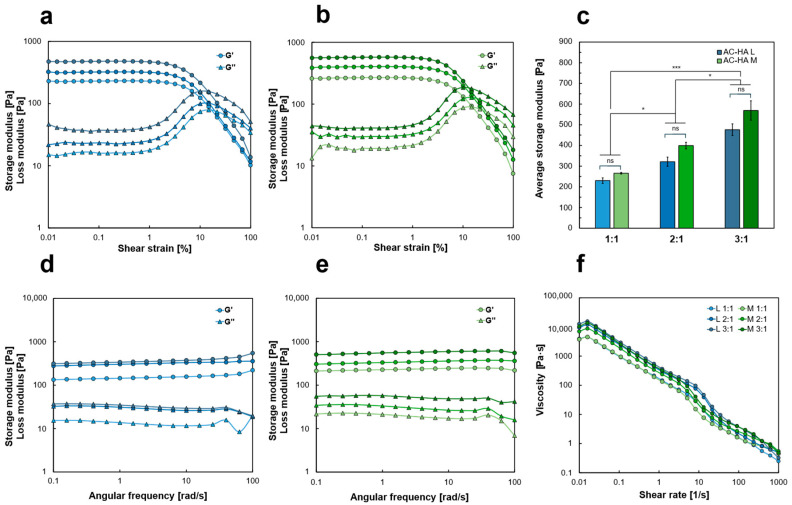
AC-HA rheological characterization. (**a**) Amplitude sweep tests for the three different dilutions tested (1:1, 2:1, and 3:1 respectively shown with increasing intensity of color) of AC-HA-L-containing (blue) and (**b**) AC-HA-M-containing (green) formulations. (**c**) Average G′ values within the LVE region of the AC-HA-L-containing (blue) and AC-HA-M-containing (green) formulations. Results presented as mean ± SD, * *p* < 0.05 and *** *p* < 0.001. (**d**) Frequency sweep tests for the three different dilutions tested of AC-HA-L and (**e**) AC-HA-M formulations. (**f**) Flow sweep tests of all the dilutions tested for AC-HA-L and AC-HA-M formulations. The values reported were evaluated with measurements run at least in triplicates.

Amplitude sweep tests demonstrated a solid-like behavior (G′ ≫ G″ within the low deformation range at 25 °C), regardless of the hydrogel formulations (Figure 3a,b). The limit of the linear viscoelastic (LVE) region was observed at approximately 0.5% for all the samples. The standard deviations are not displayed in the graph, although the presented data were characterized by a percentual standard deviation lower than 10–15%, considered as the acceptable threshold for rheological tests [42]. The average G′ within the LVE, which served as an indicator of material stiffness, increased as the dilution ratio decreased (Figure 3c) both in the case of HA-L- and HA-M-based hydrogel formulations.

The difference between HA-L and HA-M followed the same trend in swelling and rheological tests, with the G′ of HA-M being slightly higher than that of HA-L. This can be attributed to the increased crosslinking resulting from the greater number of reactive groups in the HA-M polymer chain. 

Furthermore, the test showed improved mechanical properties by lower dilution in both HA-L and HA-M formulations (*p* < 0.05). HA-L- and HA-M-containing formulations showed G′ values in the range 230–270 Pa for 1:1 dilution, 320–400 Pa for 2:1 dilution, and 470–570 Pa for 3:1 dilution, comparable to previously published values at similar HA concentrations [43].

Frequency sweep tests, presented in Figure 3d,e, showed G′ ≫ G″ over a frequency range of 0.1–100 rad/s, demonstrating the solid-like behavior and good elastic features (G′ and G″ parallel profiles over the frequency range), both for HA-L and HA-M.

AC-HA hydrogels’ viscosity has been analyzed as a function of the shear rate in the range 0.01–100 s^−1^, showing shear thinning behavior (viscosity decrease vs. shear rate), regardless for the formulation selected, as shown in Figure 3f.

### 2.3. AuNR/AC-HA Nanocomposite Hydrogel Assembly and Characterization

**Figure 4:**
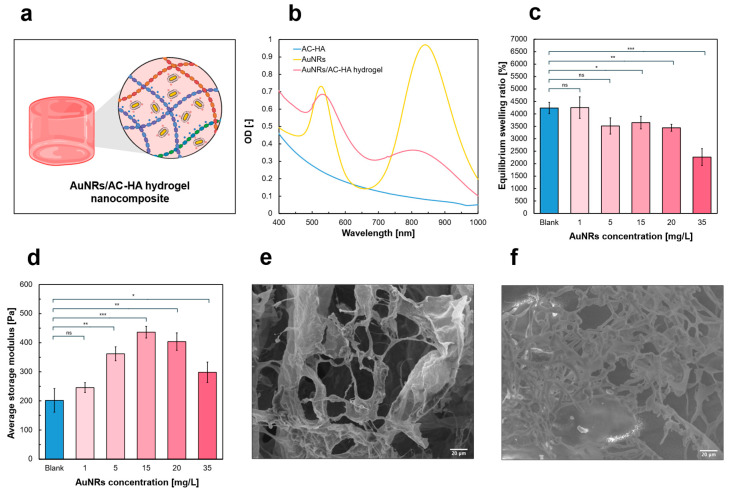
AuNRs/AC-HA nanocomposite hydrogels with AuNRs. (**a**) Schematic representation of the AuNRs-loaded hydrogels, highlighting the electrostatic interactions between AuNRs and the hydrogel three-dimensional matrix. (**b**) UV-Vis absorbance spectrum of the pristine hydrogel (light blue), the AuNRs colloidal solution (yellow), and the AuNR/AC-HA nanocomposite hydrogels (pink). (**c**) Bar chart of the equilibrium swelling ratio values and (**d**) average G′ values from amplitude sweep tests within LVE region, as a function of increasing AuNRs concentrations inside the hydrogels. Results presented as mean ± SD, * *p* < 0.05, ** *p* < 0.01, and *** *p* < 0.001. (**e**) SEM acquisitions of a gold-sputtered pristine hydrogel for morphological analysis and (**f**) non-gold-sputtered AuNRs/AC-HA nanocomposite hydrogels containing 5 mg/L AuNRs. The values reported were evaluated with measurements run at least in triplicates.

AuNRs were incorporated in AC-HA hydrogels before the sol–gel transition, during the hydrogel formulation process. The AuNRs/AC-HA hydrogel samples displayed a light pinkish color, typical of colloidal AuNRs. AuNRs remained embedded within the hydrogel matrix (Figure S4, Supporting Information) stabilized via electrostatic interaction between the AuNRs’ positively charged surface and the AC-HA negatively charged components (carbomer and hyaluronic acid) under physiological conditions [34,44], as sketched in Figure 4a.

AuNRs stability in AC-HA pre-gel solution was assessed via DLS analysis and UV-Vis over time (Figure S5, Supporting Information). The d_h_ was slightly higher in AuNRs/AC-HA than in AuNRs in water, due to the abovementioned surface interactions, likely forming a superficial polymer layer on AuNRs. On the contrary, as shown in the UV-Vis spectra, PBS caused AuNRs aggregation and precipitation, resulting in altered optical properties (as reported in Figure S5c, Supporting Information). Thus, the presence of the gel constituent polymers in PBS offered a stabilization of the AuNRs against the salt effect exerted by the PBS. The cytocompatibility of the system has been assessed in a concentration range from 1.25 mg/L to 10 mg/L AuNRs (Figure S6, Supporting Information). The analysis demonstrated that, in the presence of the AC-HA polymers, the cytotoxic effect of AuNRs was strongly reduced (~ 80% up to 5 mg/L AuNRs), likely due to the presence of HA [45]. Additionally, although the cytotoxicity of CTAB-stabilized AuNRs represents a strong limitation for translational use in real biomedical applications, in our system AuNRs are confined within the hydrogel matrix, mitigating their direct exposure to cells (Figure S4, Supporting Information). Ligand exchanges with thiol-terminated methyl-ether PEG or with sodium citrate are widespread alternatives for post-synthetical functionalization of AuNRs [46,47]. However, positively charged AuNRs surfaces should be maintained for AuNR retention within the hydrogel. UV-Vis analysis demonstrated successful AuNRs encapsulation and stability in the hydrogel, showing the longitudinal and transversal AuNRs plasmonic peaks in the UV-Vis spectrum of the AuNRs/AC-HA nanocomposite hydrogel (Figure 4b). This confirmed that the AC-HA hydrogel was able to acquire the AuNRs’ optical properties. Although AuNRs are not biodegradable, their incorporation within the hydrogel inhibits their biological migration, which may mitigate concerns related to long-term accumulation. Nevertheless, the current analyses still lack precise standardization of the technological parameters to assess accumulation, nanotoxicity, and biological effects of gold-based nanomaterials [48,49].

AuNRs aggregation within the hydrogel was excluded, as it would have resulted in a significant broadening of the plasmonic peak in the UV-Vis spectrum [50]. Additionally, AuNRs/AC-HA hydrogels were assembled by mild agitation to ensure homogeneous dispersion of the samples before gelation. Therefore, the hydrogels represented a stable support for the AuNRs, offering both stability and confinement of the AuNRs’ properties. Only the HA-L-based hydrogels with 1:1 dilution ratio were used to assess the impact of AuNRs concentration on AuNRs/AC-HA nanocomposite hydrogel properties [51]. Indeed, the key properties (swelling, rheology, gelification temperature, and kinetics) were not significantly influenced by HA molecular weight, while the dilution ratios had notable impact solely on the AC-HA mechanical properties. Moreover, the release kinetics of a drug mimetic (FITC-DXT 70 kDa) was not influenced by dilution ratio, as reported in Figure S7 (Supporting Information).

#### 2.3.1. Swelling Behavior Characterization

The swelling tests demonstrated a concentration-dependent influence of AuNRs on the nanocomposite properties, as shown in Figure 4c. The equilibrium swelling ratio was comparable to that of the blank AC-HA hydrogels for the 1 mg/L AuNRs case (~4000% equilibrium swelling ratio, with *p* > 0.05). However, the equilibrium swelling ratio decreased as the AuNRs concentration increased, with an almost halved value at 35 mg/L AuNRs compared to the pristine AC-HA sample (~2000% equilibrium swelling ratio), with a significant difference (*p* < 0.001). The swelling ratio decrease is likely due to AuNRs’ steric hindrance and electrostatic interactions, acting as a screen for polymer electrostatic repulsion [52,53]. However, the swelling kinetics were independent of the AuNRs’ concentrations, demonstrated with the equilibrium swelling ratio reached after around 5h (Figure S8a, Supporting Information), as per the blank hydrogel formulations. The swelling dependency on the AuNRs’ concentration is consistent with previous literature [51].

#### 2.3.2. Rheological Characterization

Rheological amplitude sweep tests of AuNRs/AC-HA nanocomposite hydrogels, with the AuNRs concentration ranging from 1 mg/L to 35 mg/L, demonstrated a G′ concentration-dependent behavior (Figure 4d). 

Even a low amount of AuNRs (1 mg/L) in the formulation caused an increase in average G′, compared to the pristine AC-HA hydrogel (*p* > 0.05). The average G′ reached a maximum at 15 mg/L AuNRs (almost doubled compared to the pristine hydrogel, with G′ = 436 Pa), then decreased at higher AuNRs concentrations (20 mg/L and 35 mg/L). The observed behavior might be attributed to the electrostatic interactions occurring within the hydrogel matrix and the AuNRs. At AuNRs concentrations below 15 mg/L, the AuNRs behaved as mechanical property enhancers (*p* < 0.001), acting as additional crosslinking points due to attractive forces. Conversely, at higher concentrations, the AuNRs hinder polymer chain entanglement due to increased steric hindrance and repulsive interactions [54]. Additional characterizations have been performed at 1.8 mg/L AuNRs, showing comparable behavior with the blank AC-HA hydrogel (Figures S9–S11, Supporting Information). In this regard, 1.8 mg/L AuNRs represents the maximum observed concentration for which no significant alterations were observed, for both the hydrogel mechanical and swelling properties.

#### 2.3.3. Morphological SEM Characterization

SEM imaging revealed that low concentrations of AuNRs (5 mg/L) did not significantly alter the internal morphology or structure of the hydrogel porous matrix (Figure 4e). AuNRs were not visible in the SEM images, due to the small size of the AuNRs compared to the instrumental resolution and their low concentration, nor at the higher concentrations considered in the study. Similarly, through energy-dispersive X-ray (EDX) analysis associated with the SEM, elemental gold was not detected within the samples prepared without gold sputtering (see Section 4.3.4), likely due to the extremely low concentration of AuNRs in the formulations (Figure 4f).

Although concentrations higher than 35 mg/L AuNRs would probably be visible in the SEM analysis, those concentrations were out of the scope of this work. Indeed, the intent was to provide minimal influence on all AuNRs/AC-HA nanocomposite properties compared to pristine AC-HA, by employing low AuNRs concentrations.

Accordingly, increasing amounts of AuNRs have already resulted in modified swelling and rheological properties of the composite hydrogel in the presented analyses.

### 2.4. AuNR/AC-HA Nanocomposite Hydrogels’ Light-Induced Photothermal Performances

**Figure 5:**
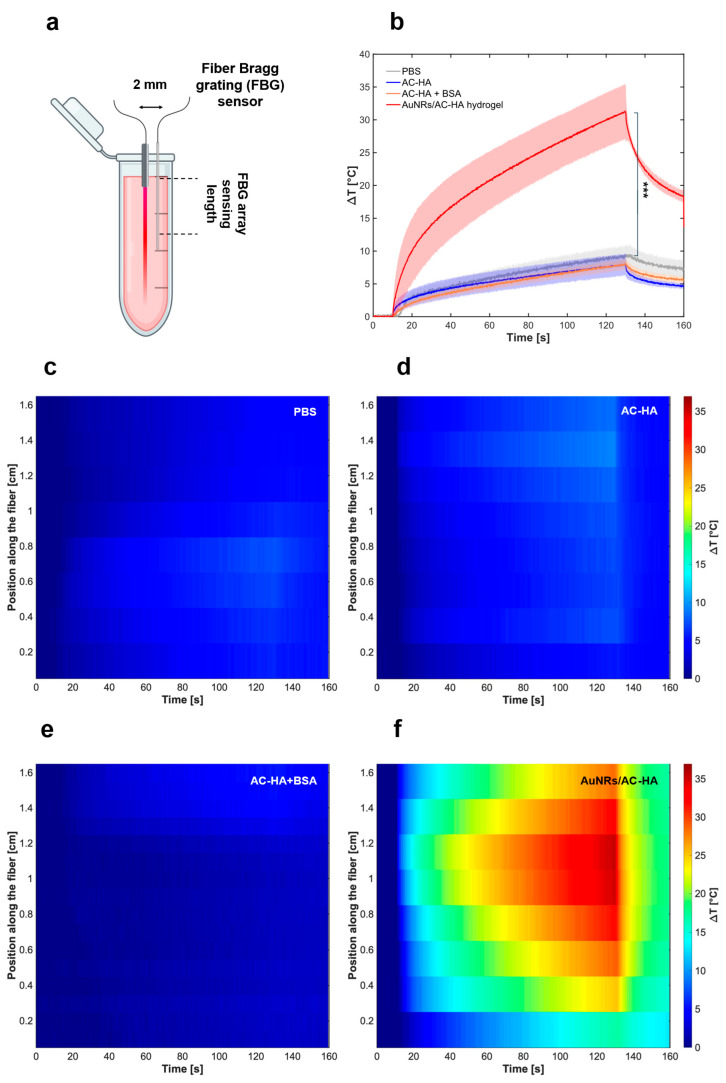
Photothermal performance evaluation of AuNRs/AC-HA nanocomposite hydrogels. (**a**) Schematic representation of the setup used for light-induced thermal heating analysis. (**b**) Maximum temperature evolution profiles of PBS (grey), AC-HA pristine hydrogel (blue), BSA-loaded AC-HA hydrogel (orange), and AuNRs/AC-HA nanocomposite hydrogel (red) with 1.8 mg/L AuNRs under NIR laser irradiation (808 nm) at a power of 5 W measured through FBG sensor arrays. Results presented as mean ± SD, *** *p* < 0.001. (**c**) Thermal maps of the spatiotemporal heat evolution in the samples evaluated during the NIR laser irradiation of PBS, (**d**) pristine AC-HA hydrogel, (**e**) BSA-loaded AC-HA hydrogel, and (**f**) AuNRs/AC-HA nanocomposite hydrogel with 1.8 mg/L AuNRs. The values reported were evaluated with measurements run at least in triplicates.

AuNR/AC-HA hydrogels’ light-induced photothermal performances have been studied at a low concentration of AuNRs (1.8 mg/L) and fixed NIR laser power (5 W), selected from a prior study [15]. This AuNRs concentration ensured the AuNR/AC-HA nanocomposite properties closely resembled those of the pristine AC-HA hydrogels, based on the previous findings. Laser delivery was fiber-based and localized in confined volumes. The ΔT induced by a CW 808 nm laser irradiation of AuNRs/AC-HA was characterized using a series of FBG sensors placed in close vicinity to the laser tip. This technology was optimal for AuNR/AC-HA nanocomposite thermal monitoring, allowing for precise recording of local thermal increase along the sample’s length, as schematized in Figure 5a. Indeed, the ΔT has been evaluated at multiple probing points, providing precise data on the thermal gradient along the irradiation direction. The FBG sensors offered key advantages compared to the different thermometric methods, including low electromagnetic interference and quasi-distributed probing points [16]. Furthermore, they allowed for direct internal temperature measurements, eliminating laser absorption artefacts (such as laser absorption by sensor external metallic components, e.g., in thermocouples) or surface-based estimations, such as for thermal camera imaging [55]. This represented a key advantage to provide a precise correlation between the local temperature increase and the drug delivery ability of the proposed setup. The maximum ΔT (in Figure 5b) showcased a significant light-induced heating in AuNRs/AC-HA samples, for a 2 min CW irradiation (ΔT = 31.5 ± 4.1 °C), in line with literature findings using similar AuNRs-loaded agarose-based hydrogels [56,57]. The maximum ΔT was assessed in BSA-loaded AC-HA samples, confirming that BSA did not influence temperature increase (comparable behavior to blank AC-HA, *p* > 0.05), as reported in Figure 5b. Despite the considerably low AuNRs concentration, the ΔT was about four times higher than 8.1 ± 1.6 °C, observed for pristine AC-HA hydrogels and PBS (*p* < 0.001), and was consistently improved, considering similar studies where ΔT = 10 °C was reached after 40 min of irradiation [58]. It is worth noting that the ΔT values discussed in our work are the maximum reached within the AuNRs/AC-HA. The heating efficiency (HE), evaluated as per Equation (2), was approximately 288%, comparable to other systems composed of AuNRs or alternative NPs (i.e., polydopamine and copper-oxide-based NPs) under NIR irradiation at the same wavelength [59,60,61]. Even though the maximum ΔT did not reach levels associated with instantaneous thermal damage to biological tissues, relevant thermal effects could arise from this system, depending on the exposure time [61]. For this reason, the irradiation conditions (irradiation time and laser power) were selected to induce moderate and localized temperature elevations, avoiding excessive heating. It is worth noting that in the proposed system the irradiation was performed only for 2 min, which is comparable to conditions employed in recent in vitro studies involving inorganic NPs [62,63,64,65]. The two-dimensional maximum ΔT (time vs. distance along the FBG array) was analyzed during laser irradiation. A notable ΔT was observed in the thermal map of AuNRs/AC-HA samples starting at t = 10 s, represented in Figure 5f, compared to controls (Figure 5c–e), where the maximum ΔT was about 10 °C. Two-dimensional thermal mapping showcased a thermal gradient within the AuNRs/AC-HA nanocomposite, with a non-homogeneous temperature distribution of the temperature along the FBG. The highest temperature increase was detected in proximity to the laser tip (8–10 mm), showing a spatial decay away from the applicator. This indicated that the local peak is a comparative metric while other gel volumes experience lower temperatures.

The selected AuNRs loading (1.8 mg/L) and short 808 nm windows follow prior AuNRs–hydrogel work demonstrating reproducible, concentration-dependent heating and are consistent with light-triggered release strategies that benchmark performance under short, localized thermal stimuli [15,60]. The gradient likely resulted from energy dissipation within the sample, given by hydrogel scattering, reducing laser penetration, and low hydrogel conductivity (comparable with water) [66]. Although metallic fillers such as AuNRs could act as thermal conductivity enhancers, the low concentration employed did not result in high-density AuNRs zones [30,56]. On the other hand, both crosslinking density and polymer concentration may have influenced the hydrogel refractive index, increasing the light scattering phenomena [67]. Lastly, the shape of the 2 mL hydrogel formulated in Eppendorf tubes could additionally condition the thermal gradient generation, representing a limitation for homogeneous development of light-induced thermal heating inside the sample.

### 2.5. AuNR/AC-HA Hydrogel Nanocomposites’ Light-Induced Drug Delivery

Laser-assisted drug delivery tests were performed using the same presented experimental setup. BSA-loaded AuNRs/AC-HA nanocomposite hydrogels were formulated as schematized in Figure 5a at 1.8 mg/L AuNRs, 1 mg/mL BSA, and sample volume of 1 mL, to allow coverage with 1 mL PBS as a receiving solution for the release test. BSA was chosen as a protein high-steric-hindrance drug mimetic for light-assisted delivery studies, owing to its dimension (66 kDa), hydrodynamic diameter (~7 nm), and well-characterized properties. For these reasons, it can act as a mimicking drug for therapeutic proteins such as: chondroitinase ABC (cABC), used for spinal cord injury treatment, fetuin-A and multiarm avidin (mAv), investigated for osteoarthritis treatment, other type IIa therapeutic proteins employed in inflammatory disease treatment, or monoclonal antibodies (mAbs) [68,69,70,71]. BSA-loaded AuNRs/AC-HA hydrogels were irradiated for 2 min at three different time points: t_0_ = 0 h, t_1_ = 0.5 h, and t_2_ = 1 h. The amount of BSA released was quantified via SDS-PAGE to avoid potential interference with optical detection after laser irradiations [72].

**Figure 6:**
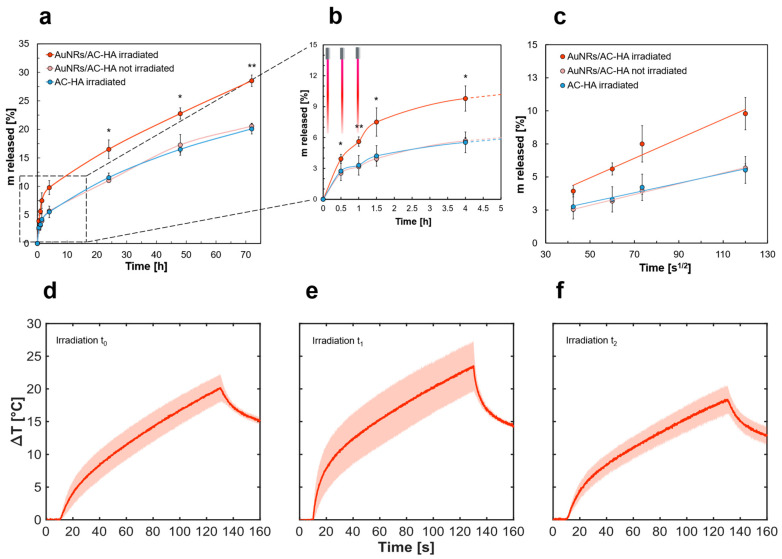
AuNRs/AC-HA nanocomposite hydrogel laser-assisted drug delivery tests. (**a**) Cumulated BSA mass released under laser-assisted tests at 808 nm wavelength with 5 W laser power from a AuNRs/AC-HA hydrogel under laser irradiation (red), AuNRs/AC-HA hydrogel without irradiation (pink), and AC-HA hydrogel under laser irradiation (blue), and (**b**) magnification of the cumulated BSA release curve within the first 5 h of release test, under the three 2 min irradiation cycles performed respectively at time 0 h, 0.5 h, and 1 h. Results presented as mean ± SD, * *p* < 0.05 and ** *p* < 0.01. (**c**) Evaluation of the cumulated release of BSA from AuNRs/AC-HA hydrogel under laser irradiation (red), without laser irradiation (pink), and from an AC-HA hydrogel under laser irradiation (blue) against the time square root, indicating a Fickian diffusion (*p* < 0.0001). (**d**) Temperature evolution profiles of AuNRs/AC-HA hydrogel nanocomposite loaded with BSA under NIR laser irradiation (808 nm) at a power of 5 W performed at different time points, respectively time 0 h, (**e**) time 0.5 h, and (**f**) time 1 h. The values reported were evaluated with measurements run at least in triplicates.

The cumulated mass of BSA released in the presence of AuNRs under laser irradiation slightly exceeded that of the control samples (Figure 6a).

The irradiation effect was visible during the initial 1.5 h of the release tests, with a steeper slope between the irradiation time points compared to the case of the non-irradiated sample (*p* < 0.05, at all the time points) as evidenced in Figure 6b (and Figure S12, Supporting Information). 

Furthermore, the irradiation effect was reflected in the equilibrium plateau reached after 72 h, where the BSA released was ~8% higher than controls (*p* < 0.01), using very short irradiations. Analysis of the electrophoresis gels and protein densitometry revealed improved release in irradiated samples, confirming the absence of BSA aggregation due to ΔT and laser irradiation (Figures S13 and S14, Supporting Information) [73]. The difference in release kinetics was visible only in the case of AuNRs-containing hydrogel, demonstrating that drug release enhancement was directly connected to the temperature increase within the polymeric matrix. The behavior was ascribed to the increased mobility and diffusivity of the BSA at the higher temperatures reached during the irradiations. Additionally, the internal hydrogel structure may be subjected to modifications due to agarose thermal responsiveness, exhibiting reversible thermal changes from 35–50 °C, according to its source and processing conditions [74]. For this reason, reversible hydrogel mesh enlargement was likely obtained, resulting in an improved BSA release, as reported in a similar study [24]. Repeated irradiation did not reveal visible macroscopic degradation, collapse, or phase separation of the hydrogel within the irradiation regimes explored. The release profile of AuNRs/AC-HA samples is characterized by low standard deviations, evidencing reproducibility of the conditions. Importantly, the release profile of pristine AC-HA hydrogel subjected to repeated irradiations is comparable to the non-irradiated samples. This further confirms that the irradiation cycles do not induce damage to the hydrogel structure, which is consistent with the temperature increases achieved under NIR exposure, below levels typically associated with irreversible hydrogel damage.

The cumulated release was plotted as a function of the square root of time (Figure 6c) to investigate the diffusion mechanism according to the Higuchi model formulation as reported in Equation (3). The three profiles exhibited a linear trend, indicative of a Fickian diffusion release mechanism, with the slope of the irradiated BSA-loaded AuNRs/AC-HA being higher than both the non-irradiated control and the irradiated BSA-loaded AC-HA pristine hydrogel. The analysis showed the enhanced diffusion rate only in the presence of AuNRs under NIR irradiation, with a reproducible and consistent response upon NIR irradiation, confirming the reliability of the light-induced modulation of the release process. The experimental data were additionally fitted with the Korsmeyer–Peppas model, described in Equation (4), to characterize release kinetics from polymeric matrices. The coefficients obtained from the mathematical fitting are reported in Table S2 (Supporting Information). The resulting values indicated a diffusion-dominated release, where the n exponent was consistently close to 0.45 for the AuNRs/AC-HA irradiated case, further confirming the Fickian-diffusion-dominated release. On the other hand, the exponent values for the case of non-irradiated AuNR/AC-HA samples and the AC-HA irradiated samples were slightly lower than 0.45, indicating a quasi-Fickian diffusion, probably given by a stronger diffusional barrier.

The maximum ΔT profiles (Figure 6d–f) proved the stability of AuNRs/AC-HA nanocomposite hydrogels’ photothermal performances under repeated CW irradiations. The observed values were lower than that of the 2 mL sample case, likely due to the reduced number of nanoheaters in AuNRs/AC-HA nanocomposite hydrogels at the same AuNRs concentration [75]. The obtained temperatures increased BSA cumulated release, maintained protein thermal stability, and ensured hydrogel stability, which could be subjected to thermal damage at temperatures above 70 °C. It is worth noting that the recorded temperatures represent the maximum ΔT reached within the sample and not the homogeneous sample temperature. Therefore, the real temperature experienced by BSA and the hydrogel surrounding it is significantly lower than the maximum temperature reached, due to thermal dissipation, as explained in the previous section.

Alternative photothermal drug delivery platforms have been reported in the literature, which employ graphene oxide or polydopamine as photothermal agents, exploiting NIR irradiation to achieve light-triggered release in different biomedical contexts [76,77,78]. However, a straightforward comparison of the performance is challenging, as the studies differ in terms of photothermal agent concentration, irradiation protocols, and setup. Consequently, differences in release enhancement cannot be directly attributed to material design alone. Compared to other systems, the present AuNRs-integrated hydrogel provides a distinct in situ temperature monitoring strategy, offering quantitative insight that is infrequently addressed in other works. The obtained experimental results showcased that a minimal amount of AuNRs in the hydrogel and short irradiation cycles enhanced both the drug delivery and photothermal performance of the nanocomposite, compared to other NPs-loaded platforms [13]. Thereby, this platform offers a foundation for advanced application of laser-assisted drug delivery systems, with the support of reliable temperature monitoring systems.

## 3. Conclusions

The incorporation of AuNRs into hydrogels has emerged as a promising strategy for developing advanced stimuli-responsive, e.g., light and temperature, DDSs for biomedical applications. In this work, we propose a nanocomposite hydrogel composed of a ternary mixture of agarose–carbomer–hyaluronic acid with AuNRs to study the light-induced photothermal and mimetic drug delivery performances of the system, aiming to correlate temperature variation with drug delivery enhancement. The core contribution of this study lies in demonstrating a material-based strategy for integrating photothermal responsiveness into chemically crosslinked hydrogels, offering a tunable and reproducible approach to control molecular diffusion under external light stimulation. Accordingly, the release experiments are intended to demonstrate controlled, light-modulated diffusion within a confined hydrogel matrix, rather than to claim therapeutic efficacy.

The AC-HA hydrogel showed tunable rheological properties according to the constituents’ ratio, whilst equilibrium swelling ratios and swelling kinetics were almost unaffected by HA molecular weight and polymer ratios. AuNRs were homogeneously encapsulated into the three-dimensional hydrogel matrix, verified by the reproducibility of the nanocomposite physico-chemical and optical properties. The proposed strategy represents a reproducible AuNRs encapsulation method within a chemically crosslinked hydrogel, without additional polymer functionalization. The AuNRs were maintained in the hydrogel matrix via electrostatic interactions with the hydrogel polymeric constituents, having a synergistic effect in hydrogel properties’ enhancement. AuNRs significantly influenced the swelling and rheological properties in a concentration-dependent manner in AuNRs/AC-HA nanocomposite, while maintaining the internal hydrogel morphology. The light-induced photothermal heating in the AuNRs/AC-HA nanocomposite under NIR light irradiation, evaluated using FBG sensor arrays, showcased a four-fold temperature increase (with HE 288%) compared to controls, with a consistently low concentration of AuNRs in the sample, further demonstrating thermal gradients, arising from low thermal conductivity and laser power scattering. Finally, the NIR-induced AuNRs/AC-HA drug delivery performances demonstrated enhancement of the BSA cumulated release, while maintaining hydrogel and protein thermal stability under repeated CW irradiations. BSA was selected as a mimetic, making it a good candidate to represent protein-based drugs, e.g., cABC, fetuin-A, type IIa therapeutic proteins, or mAbs, for disparate applications. Indeed, in the real case scenario of a light-assisted therapeutic protein delivery system, the precise temperature evaluation is crucial to assess thermally induced structural damage, as loss of therapeutic effect might arise from protein denaturation. While these results indicate that the hydrogel maintains structural and functional stability during repeated irradiations, future studies will focus on post-irradiation mechanical characterization of the system. Based on this study, the presented platform demonstrated the potential for light-assisted applications where photothermal effects can be exploited. Future investigation would benefit from the presented outcomes to select specific hydrogel and AuNRs conditions to obtain tailored properties for applications. In this perspective irradiation parameters and AuNRs concentrations can be finely tuned to optimize the therapeutic outcomes in specific biomedical applications. Moreover, the use of FBG sensors represents a key advantage in this study, as they allowed precise maximum temperature evaluation within the system, serving as an investigation platform for thermal and structural damage to potential protein therapeutics.

## 4. Materials and Methods

### 4.1. Materials

Tetrachloroauric (III) acid trihydrate (HAuCl_4_ ⦁ 3H_2_O, 99%, Sigma Aldrich, St. Louis, MO, USA), L-ascorbic acid (AA, 99%, Sigma Aldrich), sodium borohydride (NaBH_4_, 99%, Sigma Aldrich), sodium oleate (NaOL, 99%, Sigma Aldrich), silver nitrate anhydrous (AgNO_3_, >99%, Sigma Aldrich), cetyltrimethylammonium bromide (CTAB, ≥98%, Sigma Aldrich), hydrochloric acid (HCl, >37%, Sigma Aldrich), agarose (MW = 200 kDa, Invitrogen Corp., Carlsbad, CA, USA), carbomer 974P (MW = 1 MDa, Fargon, Hengelo, The Netherlands), sodium hyaluronate (HA-L, MW = 10 kDa, Lifecore, Chaska, MN, USA), sodium hyaluronate (HA-M, MW = 40 kDa, Lifecore, USA), Dulbecco phosphate buffer (PBS, Sigma Aldrich), bovine serum albumin (BSA, Sigma Aldrich), fluorescein isothiocyanate dextran (FITC-DXT, MW = 70 kDa, Sigma Aldrich), sodium hydroxide pellets (NaOH, ≥97%, Sigma Aldrich), 2-amino-2-(hydroxymethyl)-1,3-propanediol (Tris base, >98%, Sigma Aldrich), sodium dodecyl sulfate (SDS, ≥99%, Sigma Aldrich), glycine (≥99%, Sigma Aldrich), glycerol (≥98.5%, Sigma Aldrich), Dulbecco modified Eagle medium (DMEM, Gibco, Grand Island, NY, USA), L-glutamine (Euroclone, Pero, Italy), penicillin–streptomycin (Gibco), fetal bovine serum (FBS, Gibco), 3-(4,5-dimethylthiazol-2-yl)-2,5-diphenyltetrazolium bromide (MTT reagent, Invitrogen) were used. Deionized water (18.2 MΩ) was obtained from a Millipore Milli-Q purification unit. All chemicals were used without further purification unless otherwise specified.

### 4.2. Gold Nanorod Synthesis and Characterization

The synthesis of gold nanorods (AuNRs) was adapted from a previously reported protocol following the seed–growth method [79,80]. Briefly, the synthesis is divided into the preparation of two separate solutions: the seed solution and the growth solution.

Seed solution

The seed solution was prepared by stirring 1 mL of a 0.1 M CTAB solution with 43 μL of 10 mM HAuCl_4_ until reaching a yellow color. After that, 60 μL of ice-cooled freshly prepared NaBH_4_ solution (1.89 mg in 0.5 mL Milli-Q water) was added. The mixture was maintained under stirring for 30 min at 30 °C.

Growth solution

The growth solution was prepared by mixing 0.144 g of CTAB and 0.196 g of sodium oleate in 40 mL of Milli-Q water and kept under stirring at 90 °C for 90 min. After cooling the mixture to 30 °C, 40 mL of Milli-Q water and 4 mL of a 10 mM HAuCl_4_ solution were added, keeping the mixture under stirring for another 90 min at T = 30 °C. Later, 336 µL HCl 37% *w*/*w* was added, and after 15 minutes 128 μL of 0.1 M AA solution and 800 μL of 10 mM AgNO_3_ solution were added, followed by 64 μL of seed solution. After 30 s, the stirring was stopped, and the solution was maintained under reaction for 12 h at 30 °C. The synthesized AuNRs were purified with multiple centrifugation cycles to remove excess CTAB and to obtain high AuNRs purity. Briefly, 1.5 mL of AuNRs solution was placed in an Eppendorf tube and centrifuged at 13,000 rpm for 30 min, then the supernatant (1.4 mL) was removed and the AuNRs were resuspended in the same amount of Milli-Q water. Following the same procedure, the solution was centrifuged at 4500 rpm for 15 min and the supernatant, containing AuNRs, was collected. Then, the pellet was redispersed in Milli-Q water and centrifuged again at 3100 rcf for 5 min, and the supernatant was collected. All the supernatants were mixed and stored at 4 °C.

#### 4.2.1. Transmission Electron Microscopy (TEM)

The size and morphology of the synthesized AuNRs were confirmed by transmission electron microscopy (using an EFTEM Leo 912AB, at 80 kV, by Karl Zeiss, Jena, Germany). Samples were prepared by placing a 10 µL drop of AuNRs dispersion on a Formvar/carbon-coated copper grid and dried overnight. Digital images were acquired by using a Esi Vision Proscan camera charge-coupled device (Karl Zeiss, Jena, Germany) and processed for particle size evaluation using ImageJ (1.54p version) software.

#### 4.2.2. UV-Vis Analysis

The optical properties of the synthesized AuNRs were evaluated by using a UV-Vis spectrophotometer (Jasco-630 UV–Vis spectrophotometer) over a range of wavelengths from 350 nm to 1000 nm. Here, 1 mL of sample was placed in 1 cm optical length plastic disposable cuvettes and the analysis was performed at 25 °C.

#### 4.2.3. Dynamic Light Scattering (DLS) Analysis

The size and hydrodynamic diameter of the synthesized AuNPs were evaluated by dynamic light scattering using a Malvern Zetasizer Nano ZS, equipped with a 4 mW He − Ne laser operating at λ = 634 nm (backscattered angle 173°).

#### 4.2.4. Inductively Coupled Plasma Optical Emission Spectroscopy (ICP-OES) Analysis

The Au concentration of the synthesized AuNRs was determined through inductively coupled plasma optical emission spectroscopy (Optima™ 8300 ICP-OES, PerkinElmer^®^, Shelton, CT, USA) using around 1 mL of concentrated sample.

### 4.3. AC-HA and AuNR/AC-HA Hydrogel Synthesis and Characterization

The AuNRs/AC-HA nanocomposite hydrogel synthesis was adapted from a previously existing protocol, with a few modifications [15,33]. Briefly, the hydrogel consisted of agarose and carbomer 974P with the addition of hyaluronic acid (HA).

The polymer solution was obtained by dissolving carbomer 974P and hyaluronic acid (respectively HA-L with MW = 10 kDa and HA-M with MW = 40 kDa) in PBS with a final concentration of 1.6 mg/mL and 17.5 mg/mL, respectively. Then the pH was adjusted to 7.4 using a 1 M NaOH solution. The agarose–carbomer–hyaluronic acid (AC-HA) hydrogel was achieved by dissolving agarose (in a concentration of 5 mg/mL) in the polymer solution, through electromagnetic stimulation (at a 500 W irradiated power) heating at 80 °C, in a ratio of 1 min per 10 mL of polymeric solution. Then, before the sol–gel transition process was completed, the solution was diluted in a 1:1 *v*/*v* ratio with either AuNRs colloidal solution for nanocomposite samples or varying *v*/*v*% PBS ratios for the blank samples. The gelification was carried out in 1 cm diameter cylindrical molds or Eppendorf tubes, according to the specific analysis. Sterilization, challenging for hydrogels due to their high water content and polymer sensitivity to heat, was performed under UV light and no alterations in terms of physico-chemical properties were observed.

#### 4.3.1. Swelling Kinetics Test

The hydrogels’ ability to absorb and retain water was evaluated with swelling kinetics tests carried out at room temperature. Cylindrical hydrogels (1 cm diameter, 0.5 mL volume) were frozen at −80 °C for 1 h and freeze-dried right after the formulation. The initial mass w_0_ was evaluated after freeze-drying, and then each hydrogel was soaked with around 5 mL of Milli-Q water. After specific time intervals, the excess water was removed and the rehydrated mass of the hydrogel w_t_ was recorded. Then the hydrogels were covered with 5 mL of freshly added Milli-Q water. For each time point recorded, the percentual mass swelling ratio (SR) was evaluated with Equation (1) reported below, where w_t_ and w_0_ are respectively the mass of the swollen hydrogel at time t and the initial hydrogel mass at time zero.


SR [%] = ((w_t_ − w_0_)/w_0_)⋅100 (1)

#### 4.3.2. Rheological Analysis

Rheological properties of the cylindrical nanocomposite hydrogels (10 mm diameter, 1 mL volume) were investigated by performing oscillatory rheological measurements with an Anton Paar MCR 502 rheometer equipped with a parallel plate measuring system (diameter: 25 mm, plate–plate distance: 1 mm) at a fixed temperature of 25 °C.

Amplitude sweep tests

Amplitude sweep tests were carried out to determine the linear viscoelastic (LVE) region limit, the storage modulus (G′), and the loss modulus (G″) of the hydrogel, varying the shear strain amplitude according to a logarithmic ramp ranging from 0.01% to 100% at a fixed frequency of 10 rad/s.

Frequency sweep tests

Frequency sweep tests were carried out to describe the hydrogel viscoelastic behavior in the linear range by varying the frequency according to a logarithmic ramp between 0.1 and 100 rad/s, using a constant shear strain of 0.3%, within the LVE region of each sample, evaluated through amplitude sweep tests.

Time sweep tests

Time sweep tests were carried out to study the gelation kinetics of 1 mL of hydrogel reactive mixture before the sol–gel transition within 30 min using a conical plate (diameter: 25 mm, plate–plate distance: 0.5 mm).

Temperature sweep tests

Time sweep tests were carried out in a temperature range from 90 °C to 20 °C to study the gelation evolution with variation of temperature on 1 mL of hydrogel reactive mixture, before sol–gel transition.

Flow sweep tests

Flow sweep tests were carried out to determine the variation of the viscosity (*η*) upon variation of the shear rate ($\dot{\gamma }$) in an interval ranging from 0.01 s^−1^ to 100 s^−1^.

#### 4.3.3. Infrared Spectroscopy Analysis

Infrared spectra of the hydrogels synthesized were acquired using an Agilent Cary 630 spectrometer from Agilent Technologies. The spectra were recorded at room temperature in the 650–4000 cm^−1^ range at a 4 cm^−1^ resolution and 64 scans/spectra under dry nitrogen conditions. The spectra were processed and reported with baseline correction and a reversed *y*-axis.

#### 4.3.4. Morphological Characterization: Scanning Electron Microscopy Analysis

Scanning electron microscopy (SEM) analysis was carried out on gold-sputtered cylindrical (81 cm diameter, 0.5 mL volume) dry samples to determine the internal and superficial morphology of the hydrogel samples with a Zeiss Evo50 with EDS Bruker Quantax 200 microscope. The samples were formulated and immediately frozen at −80 °C to maintain the structure of the hydrogel and then freeze-dried for the analysis.

#### 4.3.5. AuNR Release Tests from AuNR/AC-HA Hydrogels

AuNR release tests were performed on AuNR-loaded hydrogel samples, prepared as described in Section 4.3. Briefly, 0.5 mL volume hydrogels were formulated with a fixed concentration of AuNRs (5 mg/L) in a cylindrical shape (1 cm diameter) and were covered with 3 mL PBS 1× in Corning^®^ 24 multiwell plates and placed at 37 °C. At specific time intervals, 1 mL of the PBS release medium was withdrawn from each plate and replaced with 1 mL of fresh PBS. The withdrawn samples were analyzed employing a Jasco-630 UV-Vis spectrophotometer at a fixed wavelength corresponding to the longitudinal plasmonic peak of the AuNR solution.

The cumulated AuNRs release [%] was evaluated, determining the amount of AuNRs released using a calibration curve, obtained using the Lambert–Beer law, which correlates the read absorbance to the sample concentration.

#### 4.3.6. FITC-DXT Release Tests from AC-HA Hydrogels

FITC-DXT release tests were performed on pristine hydrogel samples, prepared as described in Section 4.3, where the PBS solution for dilution was substituted with a FITC-DXT solution. Briefly, 0.5 mL volume hydrogels were formulated with a fixed concentration of FITC-DXT (5 mg/mL) in a square shape inside 4.5 mL volume polystyrene cuvettes (Fisherbrand™ Disposable Cuvette) and were covered with 2 mL PBS at 37 °C. At specific time intervals, 1 mL of the PBS release medium was withdrawn from each cuvette, after volume homogenization by gentle mixing, and replaced with 1 mL of fresh PBS. Then, 100 µL of the withdrawn samples was analyzed at room temperature using a Tecan^®^ Microplate Reader at a fixed wavelength corresponding to the fluorophore absorptions (*λ* = 495 nm, in the case of FITC-DXT). The cumulated FITC-DXT release [%] was evaluated by determining the amount of FITC-DXT released using a calibration curve, obtained using the Lambert–Beer law, which correlates the read absorbance to the sample concentration.

### 4.4. AuNR/AC-HA Nanocomposite Hydrogels’ Light-Response Measurement

The thermal response of AuNRs-loaded hydrogels was characterized through temperature monitored NIR laser exposures. The test specimens, namely, the AuNRs/AC-HA nanocomposite hydrogel samples containing 1.8 mg/L of AuNRs and the control hydrogel samples with PBS, were placed in 2 mL Eppendorf tubes. To ensure equivalent density between test and control samples, the control hydrogels were diluted with PBS. Laser irradiation was carried out using a diode laser (LuOcean Mini 4, Lumics, Berlin, Germany) operating at a wavelength of 808 nm in continuous-wave mode. The laser power was set to 5 W, and a 300 µm diameter quartz multimode optical fiber (GSI Lumonics) was employed to deliver the laser light to the samples. Each sample was exposed to the laser for 120 s, with the beam focal spot set at the center of the sample. Temperature evolution and thermal distribution within the samples during laser exposure were tracked using quasi-distributed fiber optic temperature sensors. A fiber Bragg grating (FBG) array was positioned parallel to the laser delivery fiber at a distance of approximately 2 mm to measure temperature changes at multiple spatial points within each sample. The FBG array, provided by FiSens GmbH (Braunschweig, Germany), consisted of 9 FBGs acting as temperature sensing elements along the optical fiber. Each FBG featured a 1 mm active sensing length, with a 1 mm spacing between adjacent gratings, resulting in a spatial resolution of 2.0 mm over a total sensing length of 17 mm. When illuminated with broadband light, the FBG sensors reflect a narrow spectral band centered at their Bragg wavelength. Temperature fluctuations lead to proportional shifts in these Bragg wavelengths [16,81]. Sensor interrogation was performed using the HYPERION si255 system (Micron Optics, Atlanta, GA, USA), which offers a wavelength accuracy of 1 pm, equivalent to 0.1 °C. The temperature profile within each sample was reconstructed by analyzing the Bragg wavelength shifts. Static calibration of the sensors determined a thermal sensitivity of approximately 11.4 pm °C^−1^.

Optical signal acquisition began 10 s prior to each NIR laser exposure to fully capture the heating phase and continued for an additional 30 s after the laser was turned off to monitor the cooling phase. All recorded temperature data were analyzed using MATLAB^®^ 2024b (MathWorks, Natick, MA, USA). Heating efficiency (HE) has been evaluated with Equation (2) and expressed as the ratio of the temperature difference between the composite and the control to the maximum temperature increase in the control.


HE [%] = ((T_AuNRs/AC-HA_ − T_AC-HA_)/T_AC-HA_)⋅100 (2)

### 4.5. BSA Encapsulation Procedure in AC-HA Hydrogels

BSA was dissolved in PBS (10 mM, pH 7.4) under mild stirring for 2 h to ensure complete dissolution. Then an aliquot of BSA solution was pre-mixed with an aliquot of AuNRs colloidal solution and PBS in Eppendorf tubes, obtaining a final mixture with concentrations of 2 mg/mL and 3.6 mg/L BSA and AuNRs, respectively. The hydrogel was achieved by dissolving agarose (in a concentration of 5 mg/mL) in the polymer solution, through electromagnetic stimulation (at a 500 W irradiated power) heating at 80 °C, in a ratio of 1 min per 10 mL of solution. Then, before the sol–gel transition, the gelling solution was diluted in a 1:1 *v*/*v* ratio in an Eppendorf tube with the BSA and AuNRs solution up to a final volume of 1 mL or 2 mL according to the specific analysis.

### 4.6. Laser-Assisted BSA Delivery from AuNR/AC-HA Hydrogel

Bovine serum albumin delivery tests were carried out to study the release kinetics of high-steric-hindrance molecules from the hydrogel. Here, 1 mL of hydrogel in an Eppendorf tube was washed with PBS to remove the non-encapsulated bovine serum albumin from the gel surface. Then the Eppendorf tube was filled with 1 mL PBS, covering the gel, as release medium.

The laser tip was placed below the level of the hydrogel and 120 s irradiation cycles were performed three times after 30 min time intervals utilizing a diode laser operating in a continuous-wave (CW) mode at a wavelength of 808 nm (LuOcean Mini 4, Lumics, Berlin, Germany) at a laser power of 5 W. A 300 mm diameter quartz multimode optical fiber (GSI Lumonics, Billerica, MA, USA) was utilized to deliver the laser light to the sample during the irradiation cycles, while the temperature was monitored through an FBG array housing 25 FBGs, placed at 2 mm distances from the laser optical fiber. Each FBG has a grating length of 0.9 mm and the edge-to-edge distances between gratings were equal to 0.1 mm, resulting in a 24 mm sensing length. At specific time intervals during the laser-assisted release test, 200 µL of the release medium was withdrawn and replaced with 200 µL of fresh PBS. The release profiles obtained were fitted with the Higuchi and Korsmeyer–Peppas models, respectively described in Equations (3) and (4).


M/M_∞_ = k⋅t^1/2^ (3)

M/M_∞_ = k⋅t^n^ (4)

### 4.7. Sodium Dodecyl Sulfate–Polyacrylamide Gel Electrophoresis (SDS-PAGE) Quantification of BSA

SDS-PAGE was used as a method to determine the mass of BSA released from the hydrogels over time. For the analysis, 15 µL of the withdrawn release medium at each time steps was mixed with 5 µL of 4× Laemmli buffer (63 mM Tris-HCl, 10% glycerol, 2% SDS, 0.0025% bromophenol blue, pH 6.8). Then the samples were incubated for 5 min at 100 °C and centrifuged before being analyzed. The analysis was performed in electrophoresis apparatus filled with a Tris-glycine SDS running buffer (25 mM Tris base, 192 mM glycine, 0.1% SDS, pH 8), where20 µL of the previously prepared samples were loaded in the 15 wells acrylamide gels (5% stacking and 12% resolving). The electrophoresis run was carried out for ~2 h at a constant voltage of 100 mV. Finally, the gels were submerged in a solution containing Coomassie Blue for protein staining for 1 h, submerged in a destaining solution to wash out the excess dye, then rinsed with Milli-Q water. Images of the gels were acquired by a ChemiDoc Imaging System and processed using the BioRad^®^ ImageLab (version 6.1) software for integral band evaluation.

The cumulated BSA release [%] was evaluated by determining the amount of BSA released at each time point using a calibration curve obtained by running a sample of pure BSA in SDS-PAGE.

### 4.8. Cell Culture and Viability Assay

L929 cells (mouse fibroblasts) were cultured in DMEM supplemented with stable L-glutamine (2 mM), penicillin–streptomycin (100 UI/mL/100 μg/mL), and 10% heat-inactivated fetal bovine serum (FBS). Cells were cultured at 37 °C in humidified 5% CO_2_ and routinely split every 3–4 days. Cell viability was evaluated by performing a 3-(4,5-dimethylthiazol-2-yl)-2,5-diphenyltetrazolium bromide (MTT) assay. L929 cells (2 × 10^4^/well) were seeded in 96-well plates. After 24 h, cells were incubated with different concentrations of AuNRs and AuNRs/AC-HA (1.25–10 mg/L). Control cells were treated with an equivalent volume of PBS or PBS/AC-HA in medium. After 24 h of incubation, cells were treated for 4 h at 37 °C with MTT compound (5 mg/mL). Then, the MTT was carefully removed, and the cells were resuspended in acidified isopropanol (0.04 M HCl). Cell viability was determined by measuring the absorbance at 565 nm using a spectrophotometer (Infinite 200Pro, Tecan). Data were expressed as percentages of controls (vehicle) for four–five separate replicates, shown as mean ± standard deviation (SD).

### 4.9. Statistical Analysis

Analysis of variance (ANOVA) was used to analyze the experimental data obtained with Tukey post hoc tests for comparison of different groups. Statistical significance was set at a top value < 0.05. Results were presented as mean value ± standard deviation, * *p* < 0.05; ** *p* < 0.01; *** *p* < 0.001; and **** *p* < 0.0001.

## Figures and Tables

**Table 1:** AC-HA hydrogel chemical composition and formulation.

		Concentration [% m/V]		
Formulation			AC-HA	
Component	Amount	1:1	2:1	3:1
Hyaluronic acid L (10 kDa)/ M (40 kDa)	87.6 mg	0.876	1.1680	1.315
Carbomer 947 P	8.3 mg	0.083	0.111	0.125
Agarose	25 mg	0.250	0.333	0.375
PBS 1×			7.5 mL	6.66 mL

## Data Availability

The original contributions presented in this study are included in the article/Supplementary Materials. Further inquiries can be directed to the corresponding author.

## Supplementary Materials

The following supporting information can be downloaded at: https://www.mdpi.com/article/10.3390/gels12010088/s1, Figure S1: IR spectra of pristine AC-HA hydrogel; Table S1: AC-HA gelation times analyzed through inverted tube tests; Figure S2: Temperature sweep tests of AC-HA-L hydrogels and AC-HA-M hydrogels; Figure S3: Swelling kinetics comparison of the AC-HA-L and AC-HA-M hydrogel formulations respectively at 1:1, 2:1, and 3:1 dilution ratios; Figure S4: AuNRs release test from the AC-HA-L 1:1 hydrogel nanocomposite with 5 mg/L AuNRs; Figure S5: DLS comparison of AuNRs in H_2_O and AuNRs/AC-HA solution and UV-Vis analysis of AuNRs in different solutions; Figure S6: MTT cytocompatibility analysis of AuNRs at different concentrations in murine fibroblast L929 cells; Figure S7: FITC-DXT release test from the AC-HA-L pristine hydrogel sample at 5 mg/mL FITC-DXT in the gel for three different dilutions tested; Figure S8: Complete swelling kinetics of AuNRs/hydrogel nanocomposites and complete graph of the rheological amplitude sweep tests as a function of the percentual strain of the different AuNR/hydrogel nanocomposites with different AuNRs concentrations; Figure S9: Time sweep test and temperature sweep test of AC-HA-L and AuNR/AC-HA hydrogel at 1.8 mg/L AuNRs; Figure S10: Flow sweep tests of AC-HA and AuNRs/AC-HA hydrogel at 1.8 mg/L AuNRs; Figure S11: Amplitude sweep tests and swelling kinetics test of AC-HA and AuNRs/AC-HA hydrogel at 1.8 mg/L AuNRs; Table S2: Parameters obtained from experimental data fitting following Higuchi and Korsmeyer-Peppas diffusion models; Figure S12: Bar graph of the cumulated BSA release test, under the irradiation cycles performed respectively at time 0 h, 0.5 h, and 1 h; Figure S13: Gel electrophoresis run of BSA release medium of an (a) irradiated sample and (b) a non-irradiated sample; Figure S14: Integral band quantification from ImageLab^®^ (version 6.1) software of the electrophoresis gels of the withdrawn PBS receiving solution samples for laser-assisted BSA drug delivery.
