# Supplementary material for: Design and Characterization of Gold Nanorod Hyaluronic Acid Hydrogel Nanocomposites for NIR Photothermally Assisted Drug Delivery
Source: Gels. 2026 Jan 19;12(1):88. doi: 10.3390/gels12010088 (PMC12840936; doi:10.3390/gels12010088)
Supplement: Supplementary file 1 [file gels-12-00088-s001.zip › gels-4082576-supplementary.pdf]

Supporting Information

Design and characterization of gold nanorods hyaluronic acid hydrogel nanocomposites for NIR photothermal assisted drug delivery

*Alessandro Molinelli, Leonardo Bianchi, Elisa Lacroce, Zoe Giorgi, Laura Polito, Ada De Luigi, Francesca Lopriore, Francesco Briatico Vangosa, Paolo Bigini, Paola Saccomandi, Filippo Rossi**

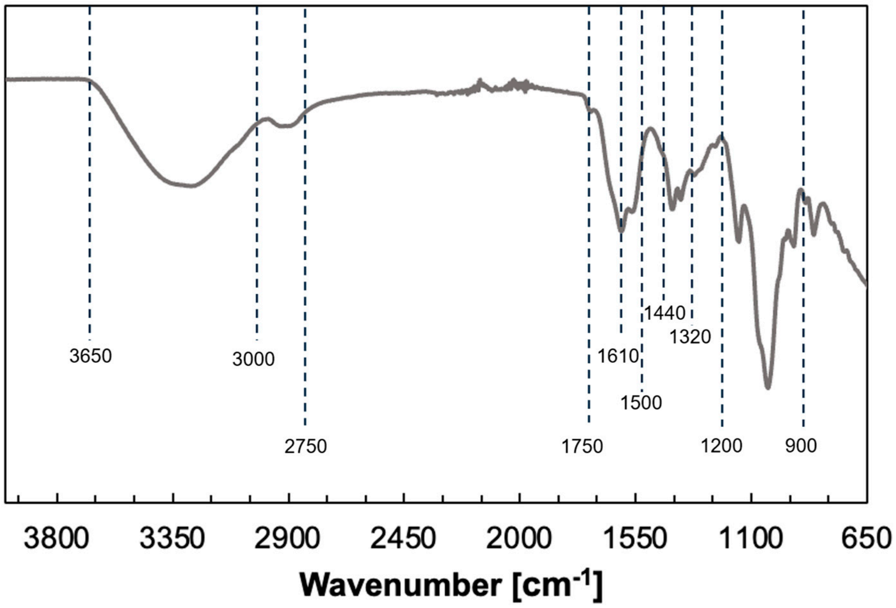

Figure S1. IR spectra of pristine AC-HA hydrogel. 3650-3000 cm^{-1}: stretching vibration of –OH bonds; 3000-2750 cm^{-1}: stretching of C-H bond; 1750 cm^{-1}: C=O stretching of ester bond; 1610 cm^{-1}: N-H stretching of amide; 1600-1500 cm^{-1}: N-H bending of amide; 1440-1320 cm^{-1}: C-O-C and C-O stretch esters and ethers; 1200-900 cm^{-1}: -C-O- stretch of alcohols.)

Table S1. AC-HA gelation times analyzed through inverted tube tests

	Gelation time [min]		
**Formulation**	1:1	2:1	3:1
**AC-HA L**	7.87	5.37	3.87
**AC-HA M**	7.25	5.10	3.37

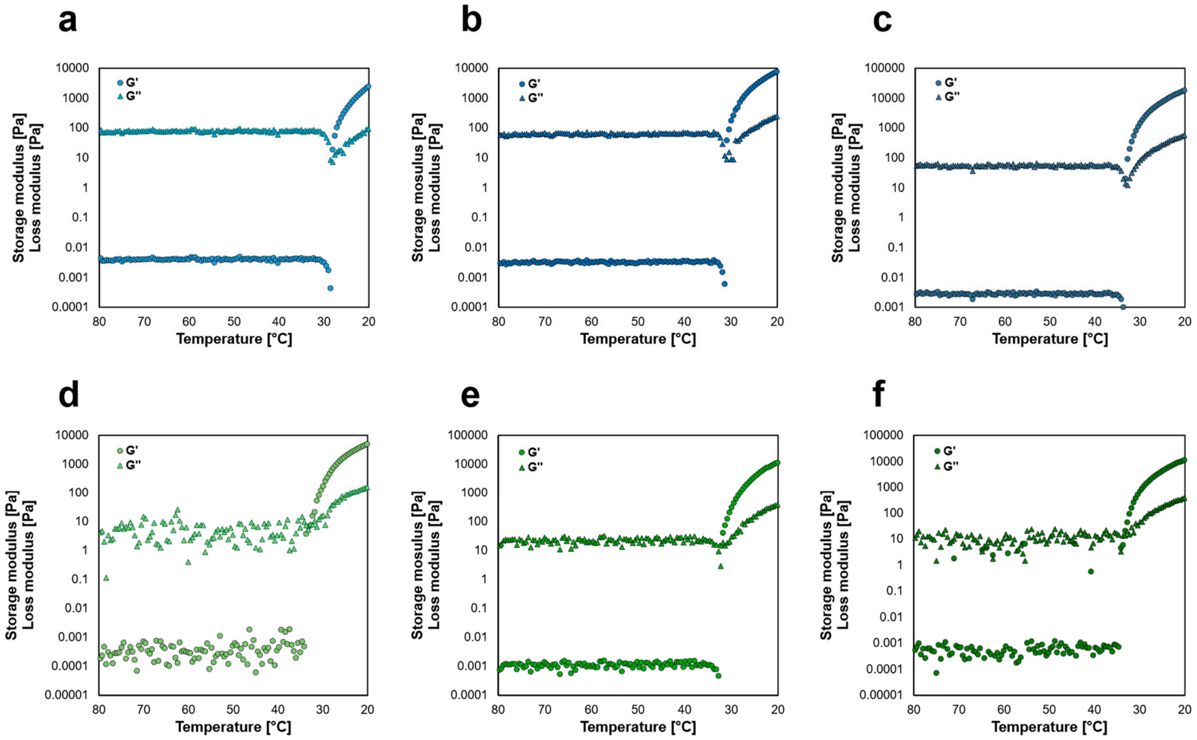

Figure S2. Temperature sweep tests of AC-HA L hydrogels at a) 1:1, b) 2:1, c) 3:1 dilution and AC-HA M hydrogels at d) 1:1, e) 2:1, f) 3:1 dilution.

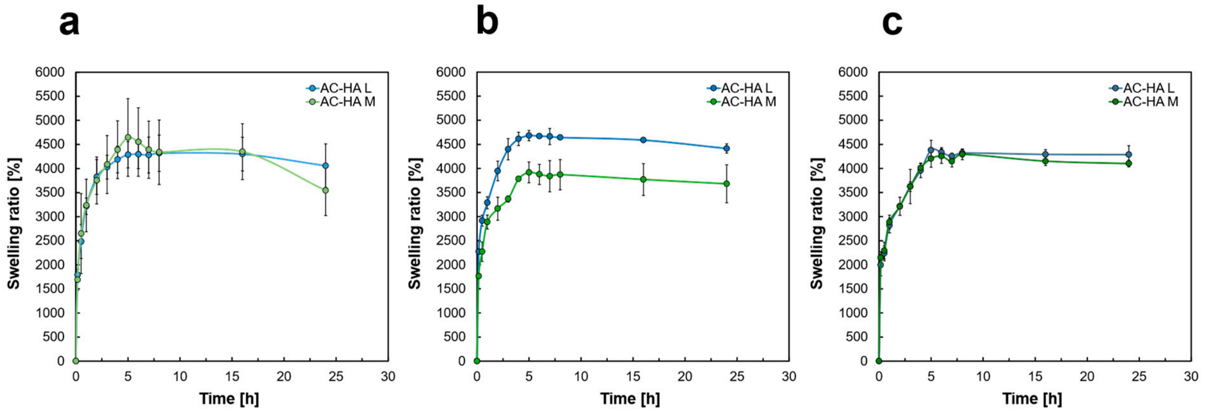

Figure S3. Swelling kinetics comparison of the AC-HA L and AC-HA M hydrogel formulations respectively at a) 1:1, b) 2:1 and c) 3:1 dilution ratio. The values reported were evaluated with measurement run at least in triplicates.

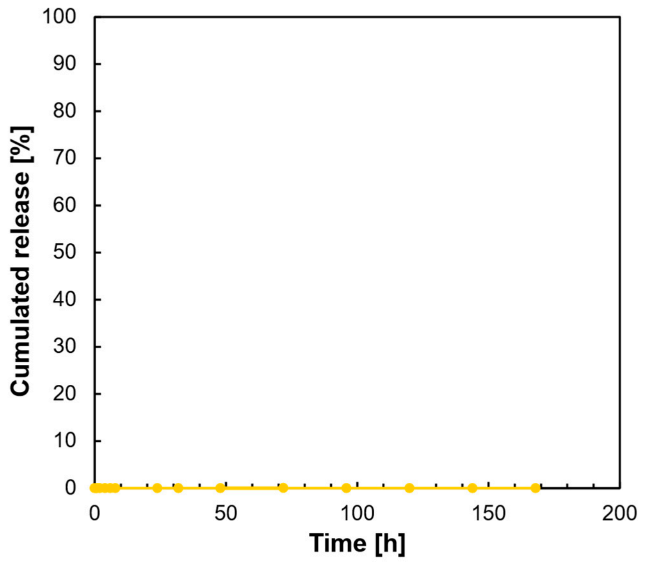

Figure S4. AuNRs release test from the AC-HA L 1:1 hydrogel nanocomposite with 5 mg/L AuNRs. The chart shows the cumulated mass of AuNRs released during time in 7 days corresponding to 168 h. The values reported were evaluated with measurement run at least in triplicates.

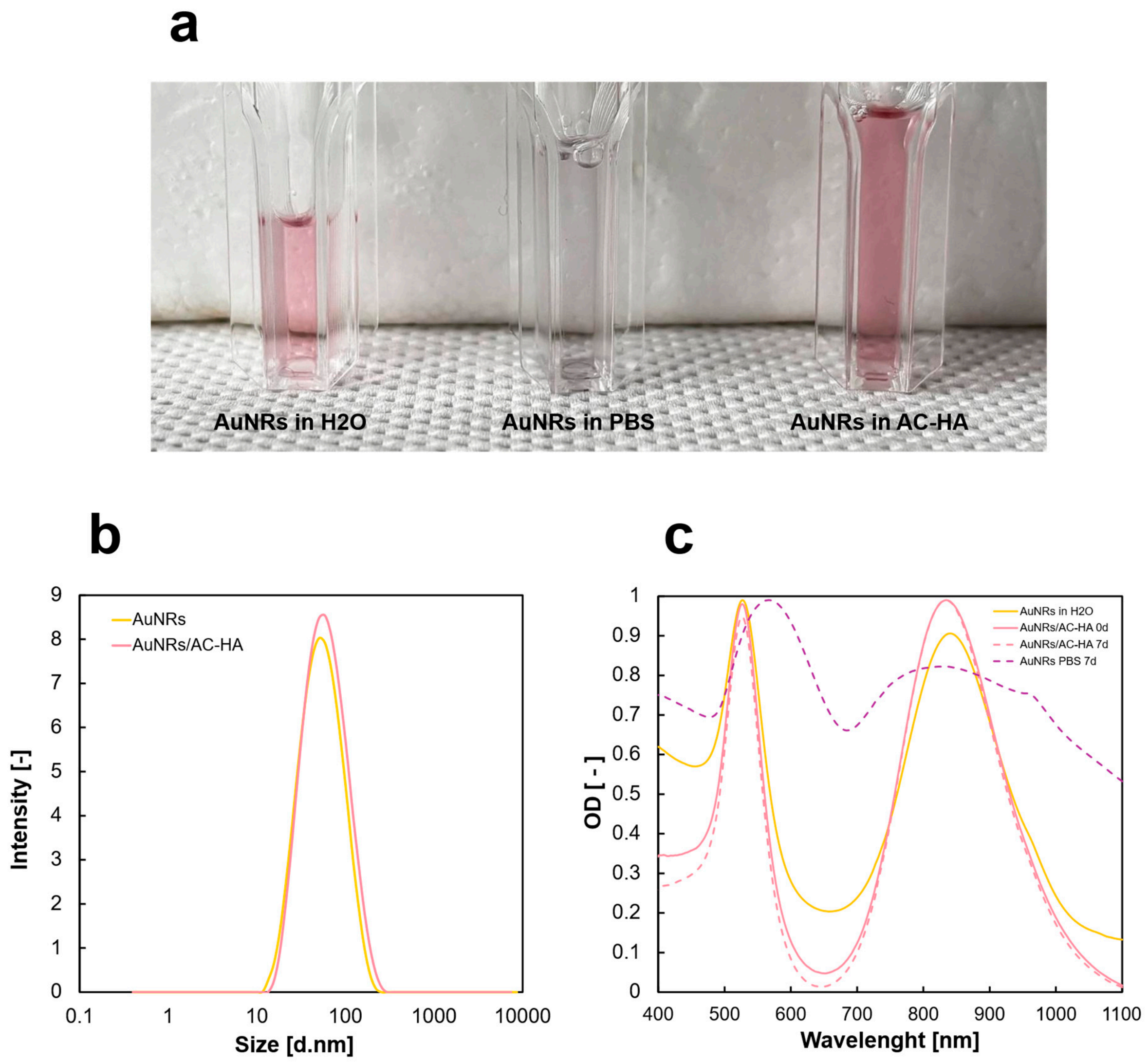

Figure S5. a) Picture of the three samples labelled. (b) DLS comparison of AuNRs in H_2O and AuNRs/AC-HA solution. b) UV-vis analysis of AuNRs in H_2O (yellow), AuNRs/AC-HA at day 0 (pink), AuNRs/AC-HA at day 7 (dashed pink) and AuNRs in PBS (dashed blue).

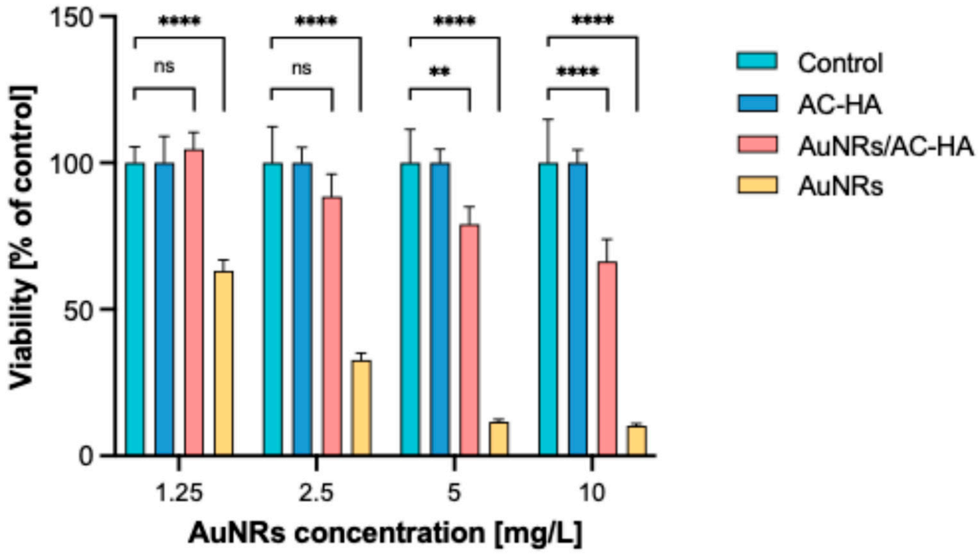

Figure S6. MTT cytocompatibility analysis of AuNRs at different concentrations in murine fibroblasts L929 cells. Results presented ad mean ± standard deviation (N = 4-5). ** p< 0.01;*** p<0.001 and **** p<0.0001.

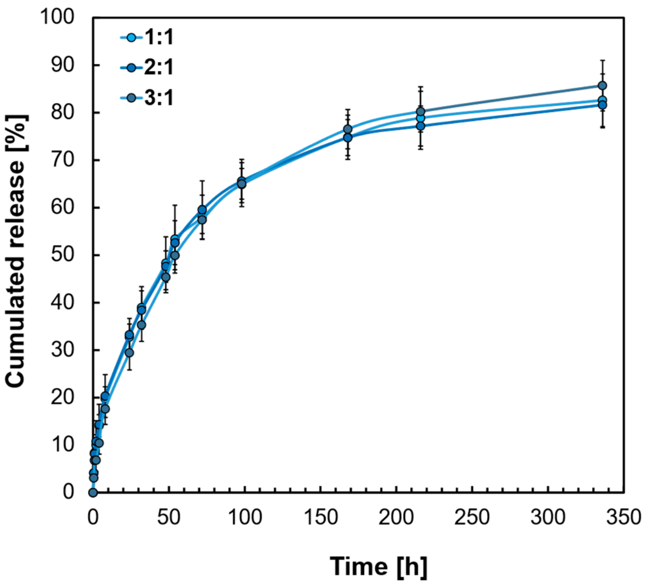

Figure S7. FITC-DXT release test from the AC-HA L pristine hydrogel sample at 5 mg/mL FITC-DXT in the gel for three different dilutions tested (respectively 1:1, 2:1 and 3:1). The chart shows the cumulated mass of FITC-DXT released during time in 14 days corresponding to 336 h. The values reported were evaluated with measurement run at least in triplicates.

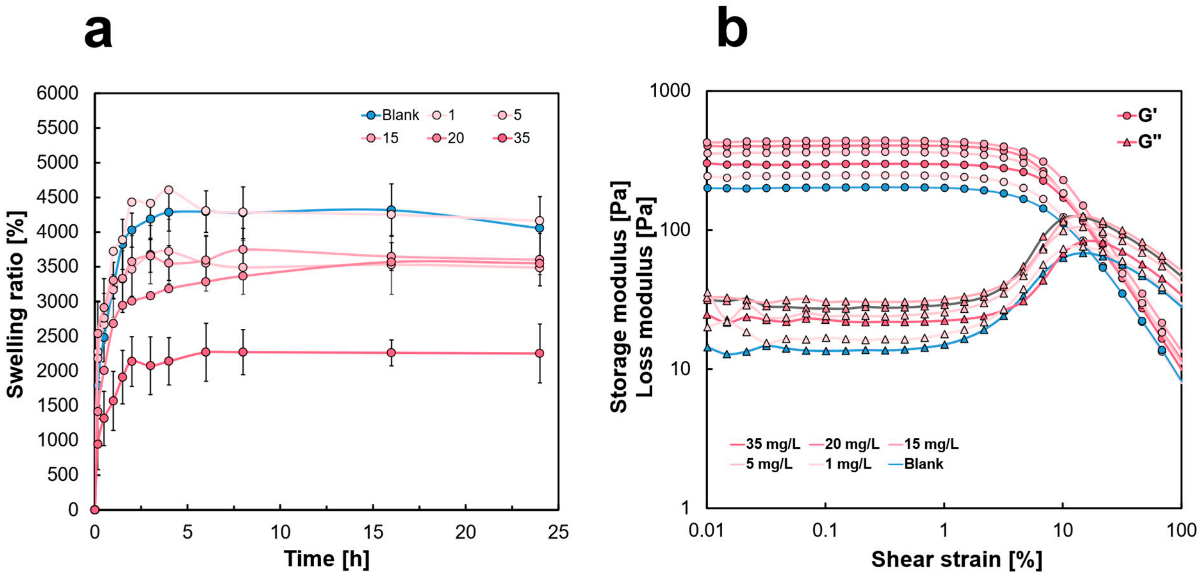

Figure S8. a) Complete swelling kinetics of AuNRs/ hydrogel nanocomposites at different concentrations of AuNRs (pink shades) and of pristine AC-HA sample (blue) all prepared with the formulation at a dilution ratio 1:1. b) Complete graph of the rheological amplitude sweep tests as a function of the percentual strain of the different AuNRs/hydrogel nanocomposite with different AuNRs concentrations. The values reported were evaluated with measurement run at least in triplicates.

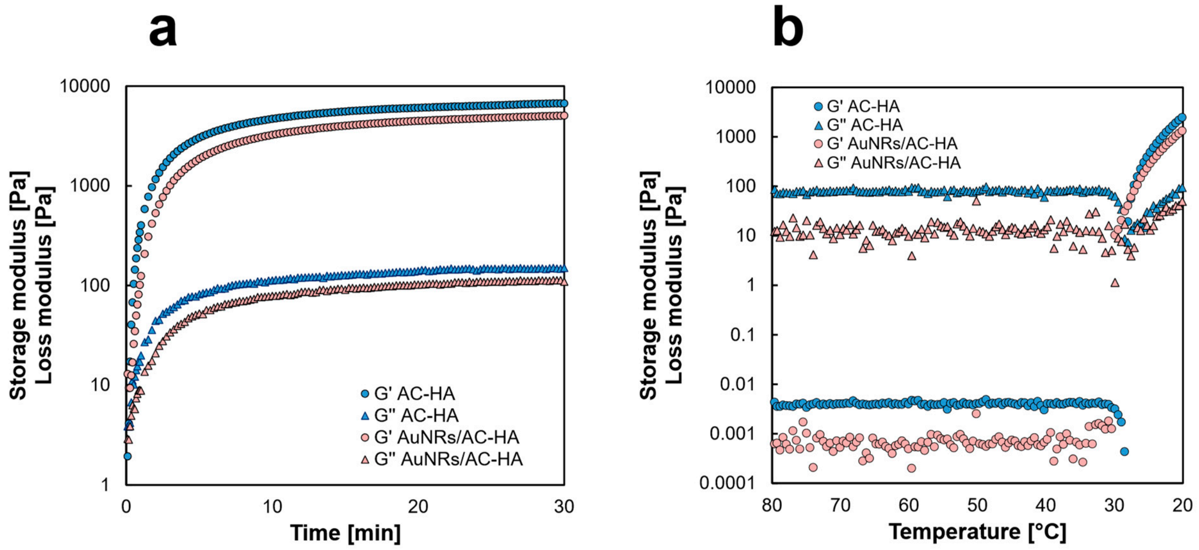

Figure S9. a)Time sweep test and b) temperature sweep test of AC-HA L and AuNRs/AC-HA hydrogel. Influence of 1.8 AuNRs on gelification kinetics and temperature crossover point of the hydrogel.

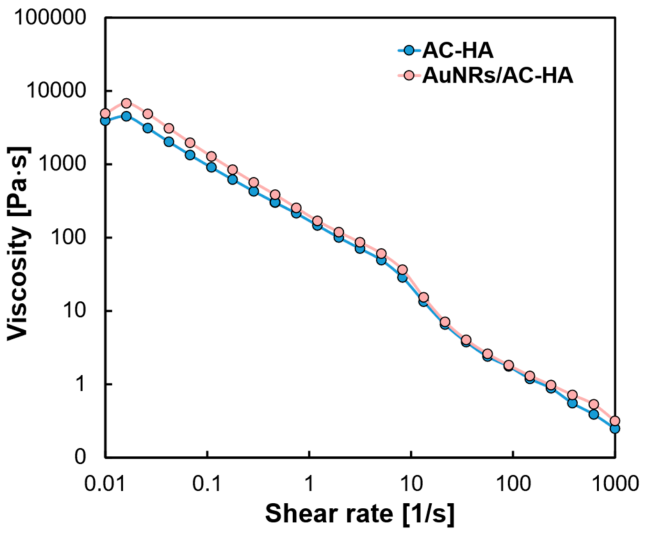

Figure S10. Flow sweep tests of AC-HA and AuNRs/AC-HA hydrogel. Influence of 1.8 mg/L AuNRs on the viscosity of the hydrogel.

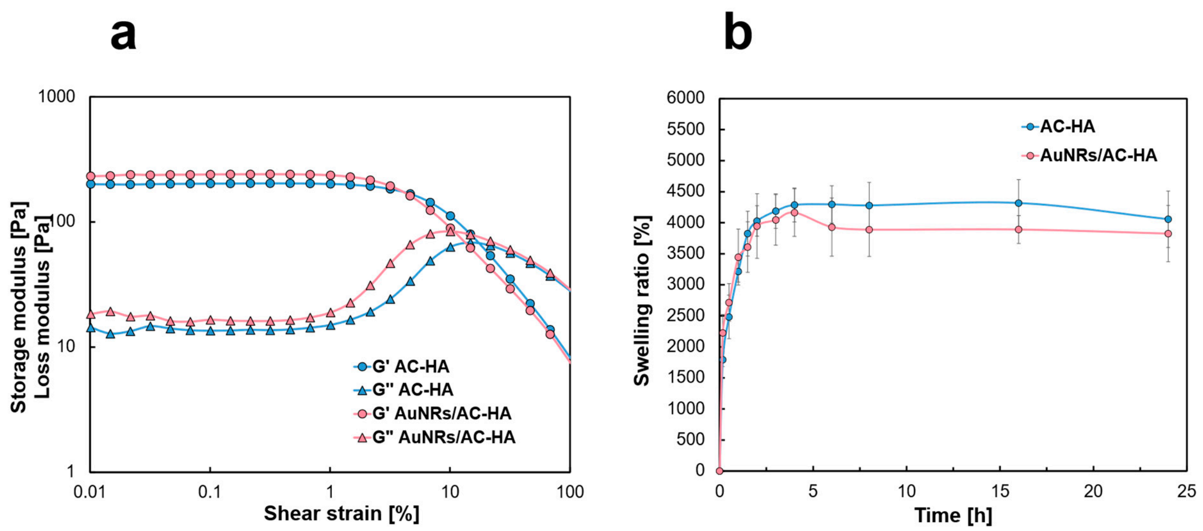

Figure S11. a) Amplitude sweep tests of AC-HA and AuNRs/AC-HA hydrogel. b) Swelling test of AC-HA and AuNRs/AC-HA hydrogel. Influence of 1.8 mg/L AuNRs on the composite properties.

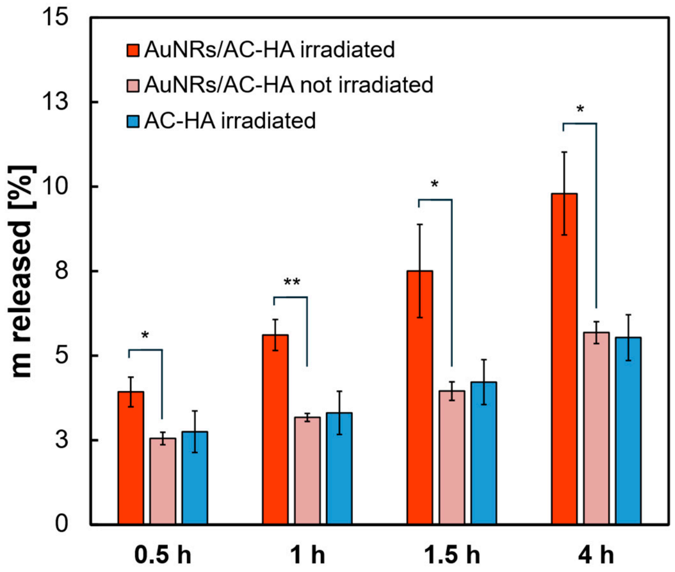

Figure S12. Bar graph of the cumulated BSA release test, under the irradiation cycles performed respectively at time 0 h, 0.5 h, and 1 h.

Table S2. Parameters obtained from experimental data fitting following Higuchi and Korsmeyer-Peppas diffusion models

	**Higuchi model**		**Korsmeyer-Peppas model**		
	k	R^2	k	n	R^2
AuNRs/AC-HA irradiated	0.088	0.99	5.613	0.44	0.96
AuNRs/AC-HA not irradiated	0.050	0.99	3.294	0.39	0.99
AC-HA irradiated	0.051	0.99	3.468	0.35	0.98

a

b

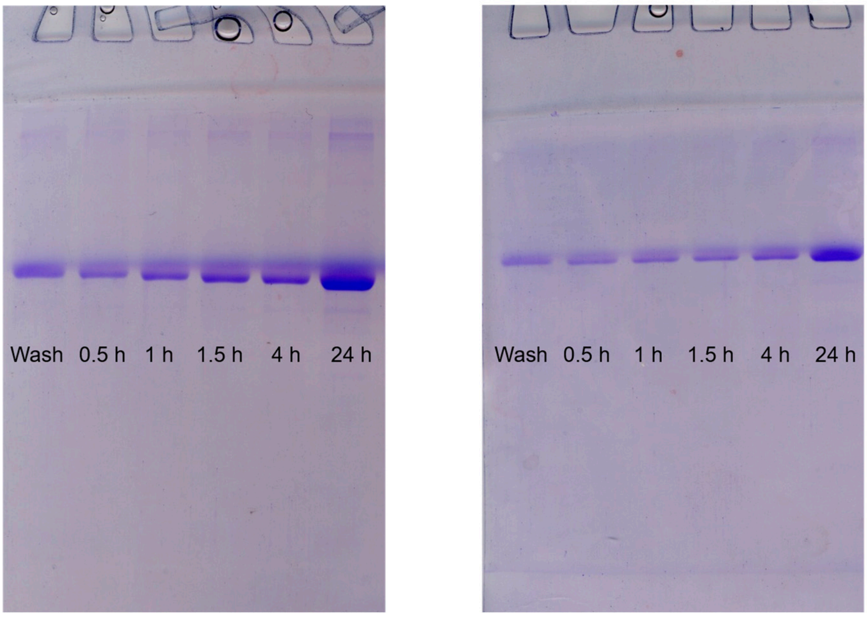

Figure S13. Gel electrophoresis run of BSA release medium of an a) irradiated sample and b) a not irradiated sample, at different time points (respectively 0.5 h, 1h, 1.5 h, 4 h and 24 h).

a

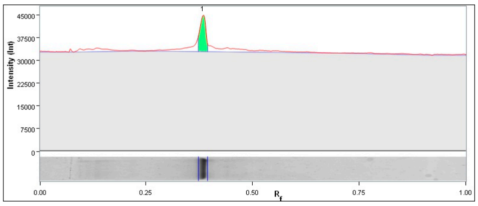

Band No.	Band Label	Mol. Wt. (KDa)	Relative Front	Adj. Volume (Int)	Volume (Int)	Abs. Quant.	Rel. Quant.	Band %	Lane %
1		N/A	0.384	48'971'754	235'733'145	N/A	N/A	100.0	29.5

Lane Background	Lane background subtracted with disk size: 10
Lane Width	5.14 mm

b

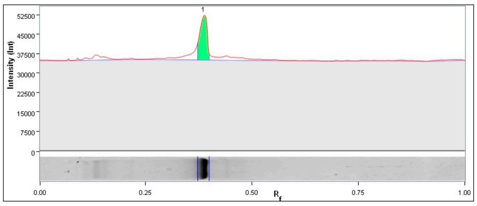

Band No.	Band Label	Mol. Wt. (KDa)	Relative Front	Adj. Volume (Int)	Volume (Int)	Abs. Quant.	Rel. Quant.	Band %	Lane %
1		N/A	0.386	80'765'334	314'680'700	N/A	N/A	100.0	41.6

Lane Background	Lane background subtracted with disk size: 10
Lane Width	5.17 mm

c

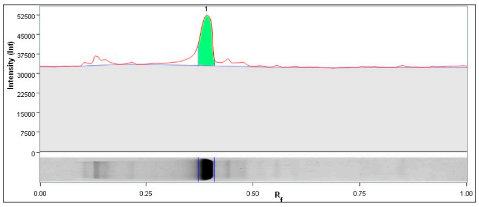

Band No.	Band Label	Mol. Wt. (KDa)	Relative Front	Adj. Volume (Int)	Volume (Int)	Abs. Quant.	Rel. Quant.	Band %	Lane %
1		N/A	0.391	162'401'750	524'639'500	N/A	N/A	100.0	49.1

Lane Background	Lane background subtracted with disk size: 10
Lane Width	5.73 mm

d

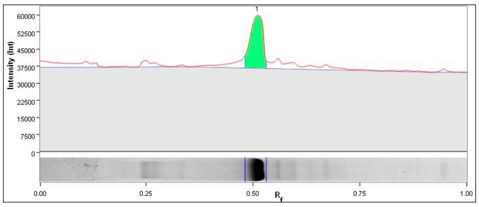

Band No.	Band Label	Mol. Wt. (KDa)	Relative Front	Adj. Volume (Int)	Volume (Int)	Abs. Quant.	Rel. Quant.	Band %	Lane %
1		N/A	0.511	205'569'570	683'677'545	N/A	N/A	100.0	43.4

Lane Background	Lane background subtracted with disk size: 10
Lane Width	5.79 mm

Figure S14. Integral bands quantification from ImageLab® software of the electrophoresis gels of the withdrawn PBS receiving solution samples for laser-assisted BSA drug delivery tests at different time points, respectively a) 1 h, b) 4 h, c) 24 h, and d) 72 h.)
